# *Glis1* and oxaloacetate in nucleus pulposus stromal cell somatic reprogramming and survival

**DOI:** 10.3389/fmolb.2022.1009402

**Published:** 2022-11-03

**Authors:** Leon Lufkin, Ankita Samanta, DeVaun Baker, Sina Lufkin, JesslynHope Schulze, Benjamin Ellis, Jillian Rose, Thomas Lufkin, Petra Kraus

**Affiliations:** ^1^ Department of Statistics and Data Science, Yale University, New Haven, CT, United States; ^2^ The Clarkson School, Clarkson University, Potsdam, NY, United States; ^3^ Department of Biology, Clarkson University, Potsdam, NY, United States

**Keywords:** intervertebral disc, nucleus pulposus, annulus fibrosus, adipose, stromal cell, 3D culture, transcriptome analysis, regenerative medicine

## Abstract

Regenerative medicine aims to repair degenerate tissue through cell refurbishment with minimally invasive procedures. Adipose tissue (FAT)-derived stem or stromal cells are a convenient autologous choice for many regenerative cell therapy approaches. The intervertebral disc (IVD) is a suitable target. Comprised of an inner nucleus pulposus (NP) and an outer annulus fibrosus (AF), the degeneration of the IVD through trauma or aging presents a substantial socio-economic burden worldwide. The avascular nature of the mature NP forces cells to reside in a unique environment with increased lactate levels, conditions that pose a challenge to cell-based therapies. We assessed adipose and IVD tissue-derived stromal cells through *in vitro* transcriptome analysis in 2D and 3D culture and suggested that the transcription factor Glis1 and metabolite oxaloacetic acid (OAA) could provide NP cells with survival tools for the harsh niche conditions in the IVD.

## 1 Introduction

**FIGURE 1:**
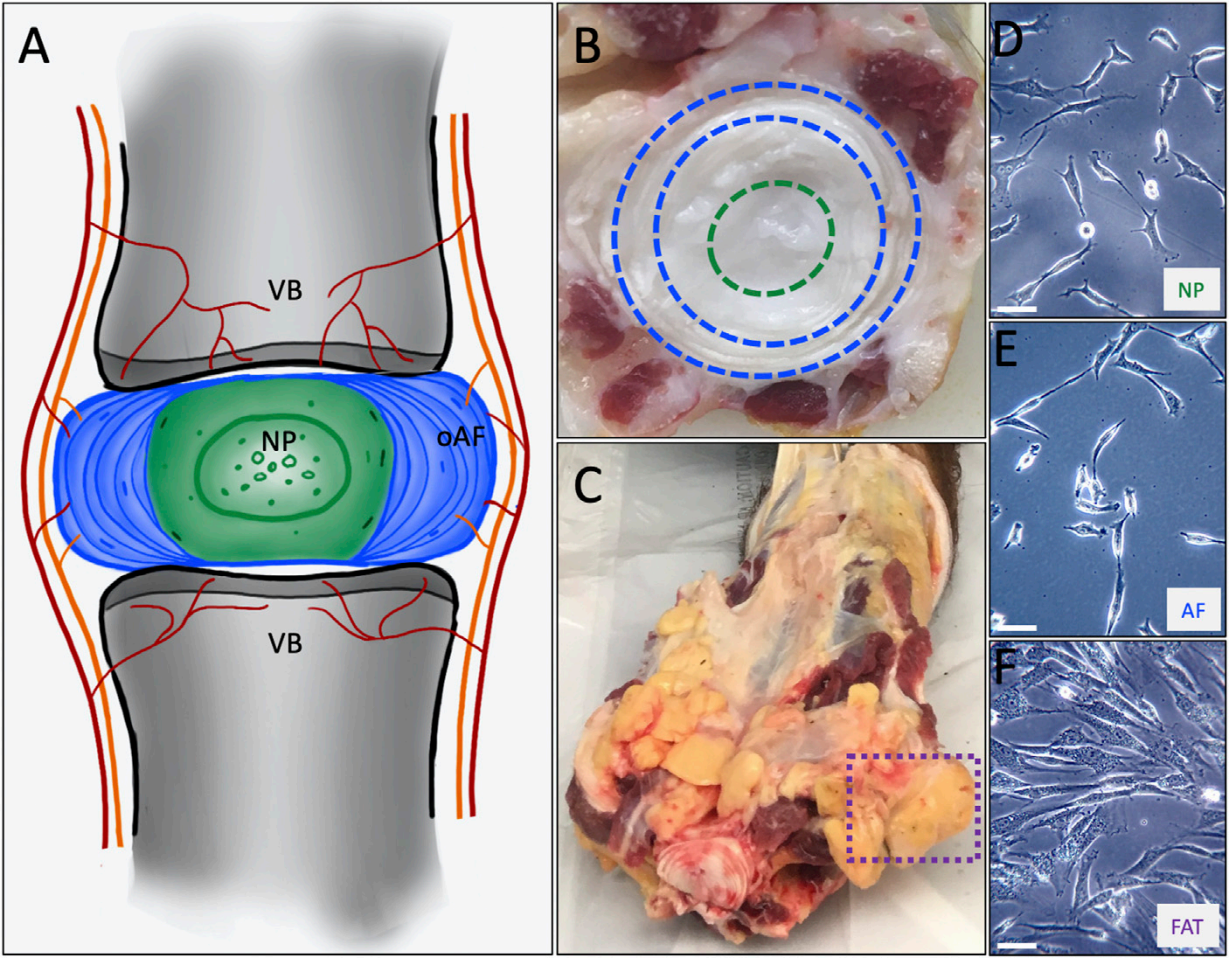
**(A)** Schematic representing the intervertebral disc consisting of an outer annulus fibrosus (oAF) and inner nucleus pulposus (NP) sandwiched between the cartilaginous endplates of the adjacent vertebral bodies (VB). **(B)** A cut through a bovine tail showing a coccygeal IVD. The outer AF as seen between the blue dashed lines and the NP within the green dashed circle served as tissue source to derive AF and NP cell lines. **(C)** The box with the dashed purple line shows coccygeal, subcutaneous adipose tissue used as source to derive FAT cells. The primary cell lines are shown at p0 as they attach to a plastic surface for NP **(D)**, AF **(E)** and FAT **(F)** cells. The scale bar represents 50 μM.

Intervertebral disc degeneration (IVDD) through accidental or lifestyle-induced trauma or aging presents a substantial socio-economic burden worldwide, affecting all genders (DePalma et al., 2011; Gatchel, 2015). As a crucial shock-absorbing organ in the axial skeleton of vertebrates, the IVD protects the spine while facilitating its mobility. The IVD consists of a central nucleus pulposus (NP) surrounded by an inner and outer annulus fibrosus (AF) and is flanked by rigid vertebral bodies and their cartilaginous endplates (CEP) (Eyre, 1979; Bayliss et al., 1988; Humzah and Soames, 1988; Oegema, 1993; Bibby and Urban, 2004; Sivakamasundari and Lufkin, 2012; Sivakamasundari et al., 2013; Bedore et al., 2014; Erwin and Hood, 2014) (Figure 1A). The NP is unique in being the largest avascular tissue among vertebrate organs. Cells in the NP of a mature IVD are sparse in numbers and embedded in vast amounts of extracellular matrix (ECM). Cells in the AF are longitudinally stretched in shape and located within the compacted, lamellar organization of densely packed ECM (Trout et al., 1982a; Kraus et al., 2017; Li et al., 2019a). In a healthy mature IVD, vasculature and innervation only reach the CEPs and outer AF, whereas NP cells depend on diffusion for nutrient access and waste product clearance (Figure 1A). Therefore, NP cells reside in a unique and naturally harsh environment, low in oxygen, reduced in nutrients (1–5 mM glucose), and slightly acidic pH of 7.1–7.4, with lactate concentrations of 16 mM in the center from lactic acid fermentation alongside proton retention by negatively charged proteoglycans (PG) in the NP-ECM. The pH drops below 7 in a degenerated disc. This harsh environment impacting the NP cell function alongside lifestyle choices, obesity, and other reasons, is considered a contributor to IVDD, eventually leading to pain and mobility impairment (Diamant et al., 1968; Nachemson, 1969; Urban et al., 1977; Bartels et al., 1998; Razaq et al., 2000; Razaq et al., 2003; Urban and Roberts, 2003; Bibby and Urban, 2004; Urban et al., 2004; Bibby et al., 2005; Wuertz et al., 2008; Wuertz et al., 2009; Gilbert et al., 2016; Wang et al., 2021a).

Regenerative medicine is a highly intricate field that proposes ways to repair degenerated tissue with minimally invasive procedures using cell-based therapies, among other strategies. The cell is the fundamental unit of all living organisms, which develops based on its genomic blueprint. Starting from a zygote, the spatiotemporally coordinated transcription of coding and non-coding DNA results in the different cell types, tissues, and organs of a multicellular organism. This regulation is modulated by transcription and signaling factors alongside a variety of non-coding RNAs discovered in recent years (Kraus et al., 2013; Kraus et al., 2019a) and epigenetic encoding of histone molecules controlling DNA access (Portela and Esteller, 2010). Differential transcription guides descendants of the initially totipotent zygote toward ectoderm, mesoderm, or endoderm. Mesoderm, the middle layer of the gastrula stage embryo, gives rise to many body cell lineages after transcriptome-dependent specialization. Terminal differentiation can eventually culminate in cell death (Kraus and Lufkin, 2017). However, some cells, referred to as stem, progenitor, or stromal cells depending on their stage of pluripotency, remain in mature tissues and retain a degree of “stemness” throughout the organism’s lifespan with a persisting degree of multipotency (Pittenger et al., 1999; Lander, 2009; Kraus and Lufkin, 2017). Based on this “stemness,” regenerative cell therapies are built.

IVDD cell therapies long engaged in the idea of using stem cells to replace ailing NP cells, increase ECM production, and restore IVD function (Wuertz et al., 2008; Sivakamasundari and Lufkin, 2013; Pennicooke et al., 2016; Kraus and Lufkin, 2017; Sakai and Schol, 2017; Xia et al., 2019; Wang et al., 2021b). Mesenchymal stem or stromal cells (MSCs) have attracted attention given their multi-over pluripotency, with the benefit of reduced tumorigenicity (Kraus et al., 2021). Among several stromal cell sources in the context of IVDD, the AF tissue would be more easily accessible than NP tissue, whereas subcutaneous adipose MSCs are an established source (Rodriguez et al., 2004; Rodriguez et al., 2005) (Table 1). The progenitor cell potential of IVD cells was suggested previously (Risbud et al., 2007; Henriksson et al., 2009; Risbud et al., 2015; Tekari et al., 2016; Thorpe et al., 2016; Kraus et al., 2017). NP cells originate from the notochord (NC) (Christ and Wilting, 1992; Choi et al., 2008; Choi and Harfe, 2011; Choi et al., 2012; McCann et al., 2012; Lawson and Harfe, 2015; McCann and Seguin, 2016), a transient embryonic signaling center in vertebrates derived from axial mesoderm. The NC was identified as the origin of NP cells through transgenic experiments in mice, identifying Sonic hedgehog (Shh), Noto, and Brachyury (Tbx1 or T-box) as important for NC cell development (Choi et al., 2008; Choi and Harfe, 2011; Choi et al., 2012). Progressive loss or trans-differentiation of NC cells in the human IVD coincides with the onset of IVDD (Choi et al., 2008; McCann and Seguin, 2016). This loss of NC cells is also seen in the mature bovine IVD but not the mature rodent or porcine IVD (Trout et al., 1982b; Urban and Roberts, 2003; Vujovic et al., 2006; Lawson and Harfe, 2015; McCann and Seguin, 2016; Kraus et al., 2017). Coccygeal bovine IVDs are, therefore, an accepted model organism to represent cells of a healthy human IVD (Li et al., 2019b). The AF is formed by sclerotome cells originating from the ventral somite and is, therefore, of mesodermal origin (Christ and Wilting, 1992; Sivakamasundari and Lufkin, 2012). Cells for tissue regeneration are typically of shared developmental origin. Among the types of mesenchymal stromal cells, adipose tissue (FAT)-derived autologous stem cells have gained popularity. Adipose cells, too, are of mesoderm origin, with various discrete deposits present in mammals (Duan et al., 2007; Berry et al., 2013). Of those, white FAT can be located as visceral fat deposits in the abdominal cavity associated with internal organs or subcutaneously. FAT-derived stromal or stem cells are a by-product of cosmetic liposuction procedures and a convenient autologous choice for regenerative cell therapy approaches (Table 1). The naturally harsh NP environment poses a challenge to cell refurbishment with any cell type, including adipose stromal cells. Metabolic studies characterized the impact of nutrients, oxygen, and pH on NP cell survival in the niche (Diamant et al., 1968; Nachemson, 1969; Urban et al., 1977; Bartels et al., 1998; Razaq et al., 2000; Razaq et al., 2003; Urban and Roberts, 2003; Bibby and Urban, 2004; Urban et al., 2004; Bibby et al., 2005; Wuertz et al., 2008; Wuertz et al., 2009; Gilbert et al., 2016; Wang et al., 2021a) and NP cells appear unusually resilient *in vitro*, even for extended culture times (Kraus et al., 2017). We show that acquired niche survival strategies distinguish NP stromal cells from AF and adipose stromal cells on a molecular level and propose that lactate metabolism is key to survival in the NP niche. These molecular adaptions could be exploited for regenerative applications.

**TABLE 1:** A recently conducted search shows applications for adipose stem cells in treating IVDD.

Clinical trials using adipose stem cells for IVDD					
Status	Name of trial	NCT number	Brief outcome	Trial phase	Publication
Completed	Adipose cells for degenerative disc disease	NCT02097862	Safe. Significant improvements in flexion, pain ratings, visual analog scale, no placebo group, no MRI or scan data	Not applicable	Comella et al. (2017)
Withdrawn	Autologous adipose tissue-derived mesenchymal stem cells transplantation in a patient with lumbar intervertebral disc degeneration	NCT01643681	Withdrawn prior to enrollment	Not applicable	Not available
Unknown	Autologous adipose-derived stem cell therapy for intervertebral disc degeneration	NCT02338271	60% with significant improvement in visual analog scale, pain score, Oswestry disability index score, and disc rehydration based on ADC mapping	Phase I	Kumar et al. (2017)

## 2 Materials and methods

### 2.1 Tissue harvest and isolation of primary stromal cell lines

**FIGURE 2:**
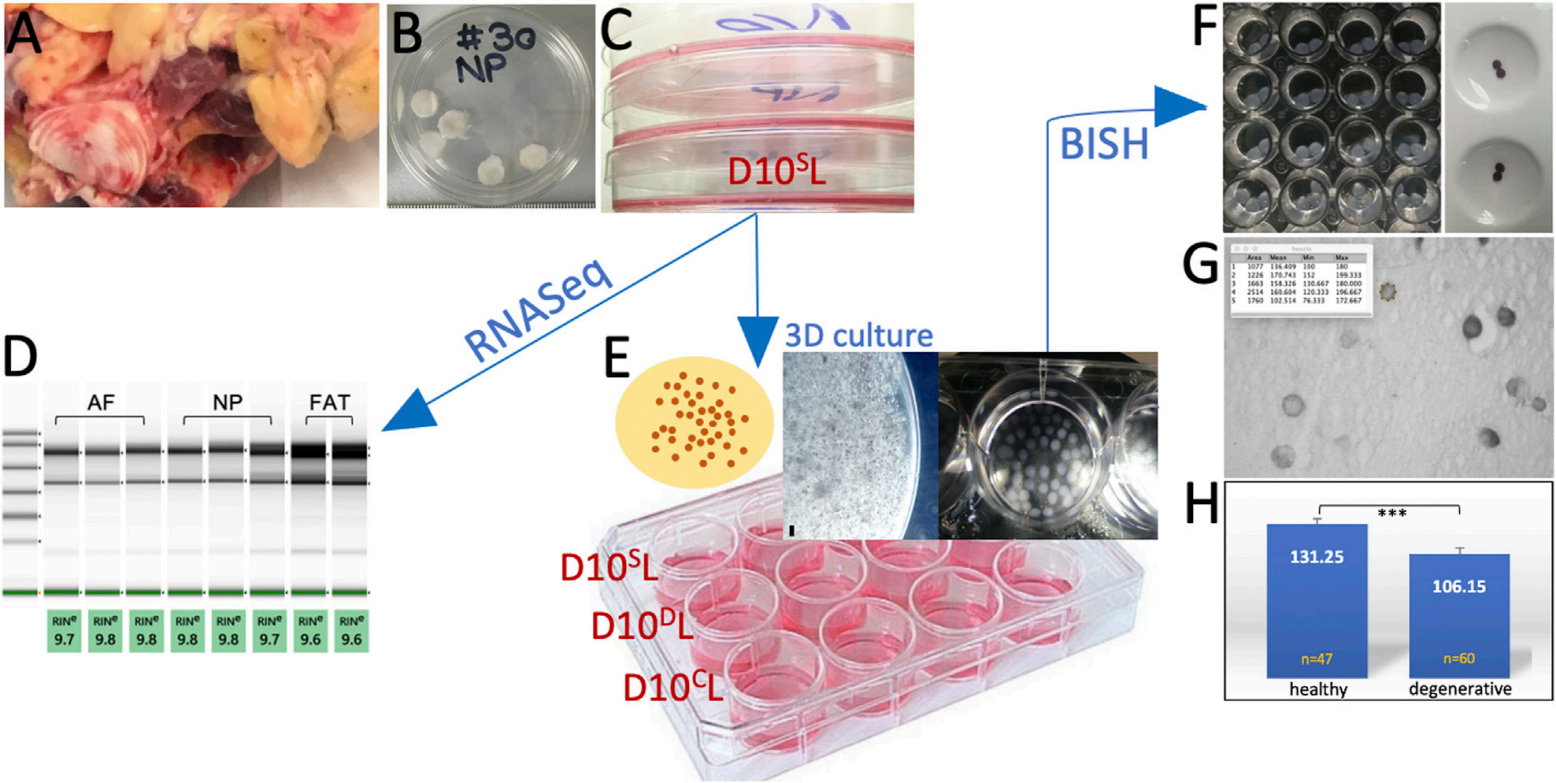
Experimental workflow showing the bovine tail as cell source **(A)**. Nucleus pulposus (NP) explants in 1X PBS prior to cell isolation **(B)**. Initial primary cell expansion in monolayer culture **(C)**. Transcriptome analysis through RNASeq after RNA quality assessment **(D)**. Cells were embedded in alginate for 3D culture as shown in the schematic. A high-magnification image of such a bead is shown next to beads collected in a 24-well plate **(E)** under standard (D10^S^L), degenerate (D10^D^L), and chondrogenic (D10^C^L) conditions. Phenotyping was carried out by RNA *in situ* hybridization on beads (BISH) **(F)**. Beads were sectioned **(G)** and submitted to ImageJ analysis **(H)**.

Cells were isolated from coccygeal bovine tissue sources of four different healthy mature animals as previously described (Kraus et al., 2017) (Figures 1B–F, 2A). Briefly, tails were retrieved from local abattoirs within 2 h of euthanasia and kept on ice until tissue dissection. After skin removal, the tissue was disinfected with a betadine solution. Subcutaneous fat, tendon, and muscle were removed before dissecting the NP and outer AF of IVDs, omitting the transition zone. Each tissue was briefly dipped into 70% ethanol (EtOH) and minced into small pieces. The pieces were moved to an untreated 35 mm polystyrene tissue culture dish (Corning/Falcon #353001) and covered with 100% heat-inactivated fetal bovine serum (HI-FBS) (Gibco # 10082147). No enzymatic treatment was applied for cell isolation. Tissue pieces were incubated for 48 h at 5% CO_2_ before the FBS was replaced with Dulbecco’s modified Eagle’s medium (DMEM) supplemented with 10% HI-FBS, 1X non-essential amino acids (MEM-NEAA) (Gibco), 1X GlutaMAX™ (Gibco), 1 mM sodium pyruvate (Gibco), 48 μG/ml gentamicin (Gibco), and 0.12 mM β-mercaptoethanol buffered with sodium bicarbonate (Gibco), thereafter referred to as DMEM with 10% FBS or standard medium (D10^S^). The medium composition was based on our embryonic stem cell work (Chatterjee et al., 2013; Sivakamasundari et al., 2017). Tissue pieces were removed after 5 days or when cells were attached to the plate (p0) (Figure 2B).

### 2.2 Cell culture

For passaging, monolayer cultured cells were rinsed with 1X phosphate-buffered saline (PBS) and disassociated with 0.05% Trypsin/EDTA (Gibco) for no more than 5 min once ∼90% confluency was reached. To assess glucose and FBS needs, primary NP, AF, and FAT cell lines from two donors were maintained in monolayer culture (Figures 1B–F, 2) supplementing glucose-free DMEM (Corning Cellgro #90-113-PB), as described earlier by adding different concentrations of glucose at 10% FBS [4.5 g/L (high (H)), 1 g/L (5.5 mM, low (L)), 0.5 g/L or 0 g/L], and different volumes of FBS [10%, 5%, 1%, and 0.2% at low (1 g/L) glucose. Three technical replicates per cell line and condition were seeded in 96-well plates at ∼300 cells/well. Once attached (0 h), cells were washed twice with 1X PBS and fixed immediately (0 h) with 4% (w/v) paraformaldehyde (PFA) or cultured for 5 days in DMEM with described glucose or FBS concentrations. Fixed cells were subjected to staining with propidium iodide following the manufacturer’s instructions (Thermo Fisher Scientific) for a total cell count using a SpectraMax i39 (Molecular Devices) multi-mode microplate reader. The ratio of average cell count was expressed as a percentage of the initial cell count (0 h). Bootstrapping was applied to test for the significance of the mean ratios of average cell counts, assuming mean counts before and after the experiment were normally distributed. The Benjamini–Yekutieli correction to control the false discovery rate (FDR) was set at 5%. Adjusted *p*-values less than 0.05 are denoted with *, those less than 0.01 with **, and those less than 0.001 with ***. Based on these observations, subsequent monolayer (2D) cell culture was carried out with DMEM/10% FBS and 1 g/L glucose (D10L) medium as the base. For 3D culture, **∼** 10^5^ cells were embedded per 1 ml alginate solution (Figure 2E) (Maldonado and Oegema, 1992; May et al., 2020). Alginate solution was prepared at 1.2% under sterile conditions by resuspending alginate powder in sterile 0.9% (w/v) NaCl until a homogeneous solution was achieved (Gilbert et al., 2016) and filtered through a 0.45-μm syringe filter. The alginate/cell solution was dripped into sterile 102 mM CaCl_2_ under agitation using a 21^1/2^-gauge needle. After curation, the beads were rinsed twice with sterile 0.9% NaCl and transferred to a 6-well dish with D10L-based culture medium for 10 days, changing the medium after 5 days (Figure 2E). D10L medium was modified to achieve three different media conditions: pH 7.1 (normal, D10^S^L), supplemented with 50 μG/ml ascorbic acid and 40 μG/ml proline (chondrogenic, D10^C^L) (Andriamanalijaona et al., 2008; Kraus et al., 2017), or adjusted to lactic acid concentration based degenerate conditions (∼pH 6.5, D10^D^L) (Nachemson, 1969; Razaq et al., 2000; Wuertz et al., 2009; Gilbert et al., 2016; Wang et al., 2021a) using 3 μl/ml lactic acid (13.4 M, Sigma) to a final concentration of ∼40 mM based on initial pH measurement. Induced pH changes remained steady during incubation based on color changes of the pH indicator phenol red. All cell culture was carried out under atmospheric O_2_ and 5% CO_2_.

### 2.3 RNA extraction and RNA sequencing

NP, AF, and FAT primary cell lines were derived from the same donor to minimize genetic bias. To minimize technical bias triplicates from NP and AF and duplicates from FAT, tissue-derived cell lines were cultured and handled simultaneously in monolayer culture using D10^S^L media as described. RNA extraction was carried out at passage 2 (p2) from ∼10^6^ cells per sample with the RNEasy Micro Kit (Qiagen) following the manufacturer’s instructions (Figure 2E). All RNA was stored at −80°C. RNA concentration was determined with a NanoDrop^®^ spectrophotometer (Thermo Fisher Scientific), and RNA quality was assessed with an Agilent 2,100 Bioanalyzer (Agilent Technologies Inc.). RNASeq was carried out by Genewiz to identify significantly differentially expressed genes from total RNA (500 ng) of three biological replicates per NP and AF sample and two biological replicates per FAT sample as per the Genewiz recommendation. RNA sequencing libraries were prepared using the NEBNext Ultra RNA Library Prep Kit (NEB) following kit protocol, fragmented, reverse-transcribed, and ligated to universal adapters, followed by index addition and library enrichment via limited-cycle PCR. The sequencing libraries were validated on an Agilent TapeStation and quantified with a Qubit 2.0 Fluorometer and quantitative PCR (KAPA Biosystems), clustered on a single lane of a flowcell and loaded on an Illumina HiSeq4000 instrument. Following 2 × 150-bp paired-end sequencing, image analysis and base calling were conducted by the HiSeq Control Software (HCS). Raw sequence data (.bcl files) generated from Illumina HiSeq were converted into fastq files and de-multiplexed using Illumina’s bcl2fastq 2.17 software. One mismatch was allowed for index sequence identification. The bioinformatic workflow followed the Genewiz protocol using GeneSCF v.1.1-p2, along with PANTHER.db and ToppFun (part of the ToppGene Suite) for Gene Ontology (GO) annotation of biological pathways, molecular mechanisms, and pathways. Graphs were generated using ggplot2 in R. ToppCluster was used for network generation of transcripts with Bonferroni correction and a *p*-value cutoff of 0.05. Generated networks were visualized and analyzed in Cytoscape.

### 2.4 RNA *in situ* hybridization

RNA *in situ* hybridisation (RISH) was used for targeted transcript-based cell phenotyping on sectioned tissue (SISH) (Kraus et al., 2001; Kraus et al., 2014; Kraus et al., 2019a; Kraus et al., 2019b), cells cultured on glass coverslips (CISH) (Li et al., 2021), or in alginate beads (BISH). Briefly, the SISH tissues from more than three donors were fixed in 4% (w/v) PFA overnight at 4°C and then washed 3 × in 1X PBS for 5 min. Samples were dehydrated through rising ethanol gradients followed by 3 × 100% ethanol and 3 × HistoClear for 10 mins each, embedded in paraffin, and sectioned at 7 μM. For CISH, coverslips containing cells of three donors were fixed in 4% PFA post-culture and subjected to the RISH procedure below. BISH cells in alginate beads from one donor were fixed in 4% PFA for at least 2 h at 4°C and briefly rinsed in bead buffer (BB) prepared as 50 mM CaCl_2_ and 150 mM NaCl in nuclease-free (nf) water, followed by 150 mM NaCl^nf^ (Figure 2F). Prior to RISH, the sections on polylysine-coated slides were de-waxed in HistoClear, rehydrated, and post-fixed with 4% PFA. Prehybridization for 2–3 h was followed by overnight hybridization, both at 62°C. Antisense digoxygenin (DIG)-labeled RNA probes based on PCR amplified templates of the bovine genome (Table 2) were *in vitro* transcribed using the DIG RNA labeling mix (Roche/Sigma), purified with RNA Spin columns, and used as 500–800 ng/ml in pre-hybridization solution (Kraus et al., 2017). Washes included Solution I (50% formamide, 5X SSC, 1% SDS; 62°C), TNT (10 mM Tris-HCl, pH 7.5, 0.5 M NaCl, 0.1% Tween 20; RT), TNT:Solution II (1:1), and Solution II (50% formamide, 2X SSC, 0.2% SDS; 58°C). Before blocking with Superblock (Thermo Fisher Scientific), samples were rinsed with 1X PBS or BB and probed with alkaline phosphatase (AP)-conjugated anti-DIG antibody diluted 2,000-fold in blocking solution. Samples were exposed overnight to an anti-DIG-AP antibody (Roche). After washes with 1X PBS or BB and 150 mM NaCl, samples were conditioned in NTMT (0.1 M Tris-HCl, pH 9.5, 0.1 M NaCl, 0.05 M MgCl_2_, 0.1% Tween 20) before the NBT/BCIP (Roche) substrate was added for color development at 4°C in the dark. All BISH experiments were performed under the same conditions for triplicates of alginate beads per probe. Beads were dehydrated as described and sectioned at 7 μM. Outcomes were documented on a Zeiss Primovert microscope (Kraus et al., 2017; Kraus et al., 2018; Amirdelfan et al., 2021). BISH data were quantified *via* ImageJ at single-cell resolution and assessed for statistically significant differences in expression levels on a population level using Student’s *t*-test. The Benjamini–Yekutieli correction to control the FDR was set at 5%. Adjusted *p*-values less than 0.05 are denoted with *, those less than 0.01 with **, and those less than 0.001 with ***. All BISH data acquisition was performed by the same researcher to avoid inter-person variability. Graphs were generated using ggplot2 in R.

**TABLE 2:** Primer sequences used to generate RNA *in situ* hybridization probes.

Gene	Primer 5′ to 3′ forward/reverse	Target/references
*Acan*	AAG​CAA​CAG​AGG​AGC​ACA​CA/AAG​CAA​CAG​AGG​AGC​ACA​CA	[11]
*Col2a1*	TCA​CAG​AAG​ACC​TCC​CGT​CT/GCA​CAA​AGC​ACA​AGC​CAG​TA	[11]
*Gapdh*	CCT​CAT​GGT​CCA​CAT​GGC​CT/TGG​TAC​ACA​AGG​CAG​GGC​TC	NM_001034034.2
*Glis1*	ATG​ACG​GAA​GCT​CGC​GCA​CC/GGC​ACG​CTT​CAA​GCC​GCA​AA	XM_010803559.2
*Gys1*	GCC​CCG​CTC​TTG​GAA​TCC​CC/TCC​GCT​CCC​AAC​TGC​AAA​CCA	NM_001101299.2
*Il1b*	CCC​CAA​AGT​CTA​CCC​CAA​GAG​G/AGA​TGC​GCC​TGC​TTC​TAG​GC	NM_174093.1
*Ki67*	CGA​GCC​TCA​GAG​CTG​AAG​TG/GAC​TGG​CTC​CGG​TTG​AGA​AG	XM_015469655.1, [57]
*Krt19*	CAG​GCG​CTG​ATC​AGT​GGT​AT/TTT​ATT​GGC​AGG​TCA​GGG​GG	[11]
*LdhA*	GTG​CAG​ATA​CAC​TTT​GGG​GGA/AGA​ACA​GTT​TAG​CAC​ATG​GCA	NM_174099.2
*LdhB*	GAG​CCT​TCC​GTG​TAT​CCT​GA/GAG​CCT​TCC​GTG​TAT​CCT​GA	NM_001316338.1
*Mdh*	AAT​CTA​GGC​ATC​GGC​AAG​GTC/GAC​CAA​GAT​TGC​AAA​GGG​GTG	NM_001013587.1
*Noto*	AGA​TGC​GGA​GTC​AGG​AAT​GG/ATG​CCA​GTT​TTG​GCA​AAG​GC	[11]
*Oct4*	GAC​ACC​TCG​CTT​CTG​ACT​TCG/CCC​TCG​GAG​TTG​CTC​TCC​AC	[11]
*P21*	GGA​AGC​ACG​TCC​TCG​TGG​GAA/GCC​CAA​CCT​TAG​AGG​GGG​CA	XM_005223326.4
*P53*	CCT​GTT​GAC​ATC​CCC​ACC​CAT/CCA​GCA​CCC​ACA​AAA​GGC​AC	X81704.1
*Pax1*	CCT​CGG​GAG​CTT​ACA​CGG​AC/AGG​AGT​GGG​AGG​GGA​CAC​TT	XM_025000893.1
*Tbox*	GCA​GTG​TTT​GAG​CGG​CAG​TC/AGC​AAA​CAT​TCT​AGC​AGG​CAG​AG	[11]
*Tnfa*	ACG​GTG​TGA​AGC​TGG​AAG​ACA/GTT​GCA​TCC​GGG​AAC​CCA​AG	NM_173966.3

### 2.5 SA-β galactosidase staining and histology

Acidic β-galactosidase staining was conducted using a staining solution of 1 mG/ml X-gal, 5 mM potassium ferricyanide, 5 mM potassium ferrocyanide, 150 mM NaCl, and 2 mM MgCl_2_ in 40 mM citric acid/sodium phosphate buffer at pH 6 (Price et al., 2002; Debacq-Chainiaux et al., 2009). The proportion of senescent, SA-β-gal-positive cells was quantitated by scoring an average of 126 cells for each condition and cell line as determined from six non-overlapping images for each condition. Data acquisition was performed by the same researcher to avoid inter-person variability. Significance was established using the proportion Z-test. The Benjamini–Yekutieli correction to control the FDR was set at 5%. Adjusted *p*-values less than 0.05 are denoted with *, those less than 0.01 with **, and those less than 0.001 with ***. Graphs were generated using ggplot2 in R. For supporting histological analysis, Mallory’s tetrachrome was used, as previously described (Kraus et al., 2017).

## 3 Results

### 3.1 Nucleus pulposus stromal cells thrive at low glucose but need FBS

**FIGURE 3:**
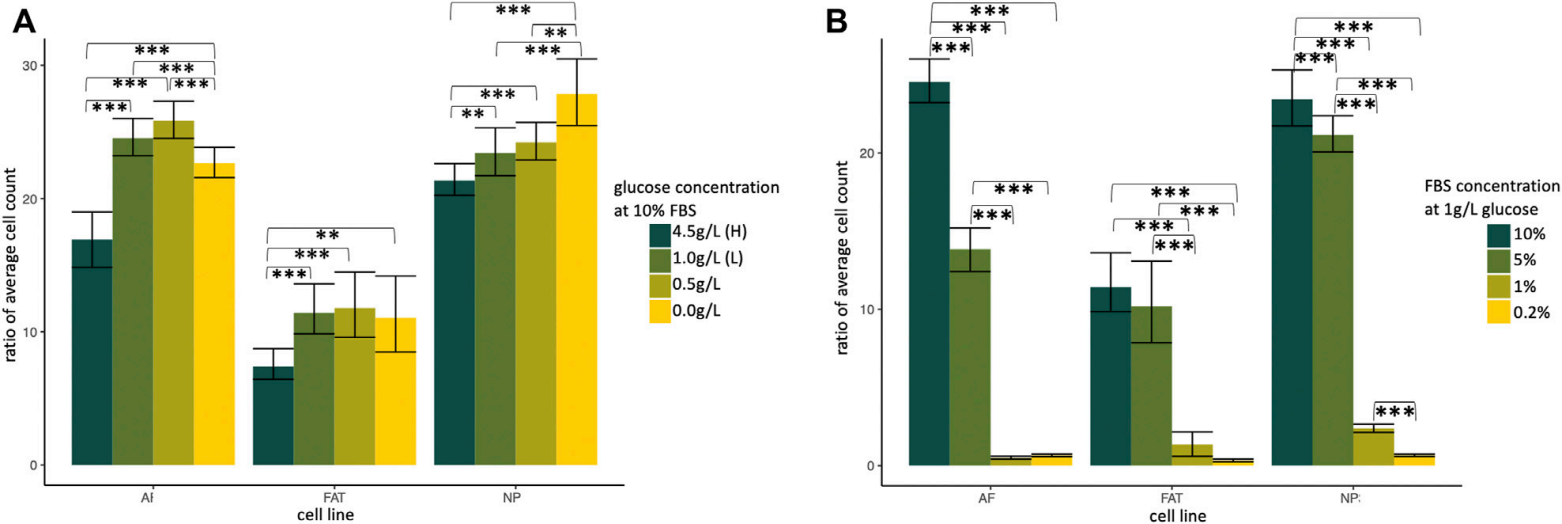
Normalized ratio of average cell count indicating cell proliferation of primary cells isolated from annulus fibrosus (AF), nucleus pulposus (NP), and subcutaneous adipose tissue (FAT). Cells were cultured in supplemented DMEM and subjected to a glucose **(A)** and growth factor **(B)** challenge. H, high; L, low; FBS, fetal bovine serum. Cells were isolated from two different donors and plated as three technical replicates for each cell line and condition.

Primary NP, AF, and FAT stromal cells derived from two donors were cultured for 5 days and analyzed as three technical replicates for different glucose or serum concentrations after normalization for the initial cell count at 0 h (Figure 3). All cell lines proliferated under reduced glucose concentrations at 10% FBS, and low glucose (1 g/L; D10^S^L) was generally tolerated well. Growth factors and other stimulants for mammalian cells *in vitro* are typically provided *via* fetal bovine serum (FBS) (Kraus et al., 2015). HI-FBS by Gibco used here contains ∼134 mg/dl glucose, which adds additional 13.4% glucose to the low glucose medium if used as a 10% medium supplement. When challenged under low glucose conditions, all cell lines tolerated an FBS reduction to 5%, with AF cells appearing most sensitive based on a total cell count. However, no live/dead assessment was conducted (Figure 3B). The results prompted us to use a low glucose medium (1 g/L) supplemented with 10% FBS (D10^S^L) as our standard to accommodate all three cell lines for the remaining experiments.

### 3.2 Transcriptome profiling of NP, AF, and FAT stromal cells

**FIGURE 4:**
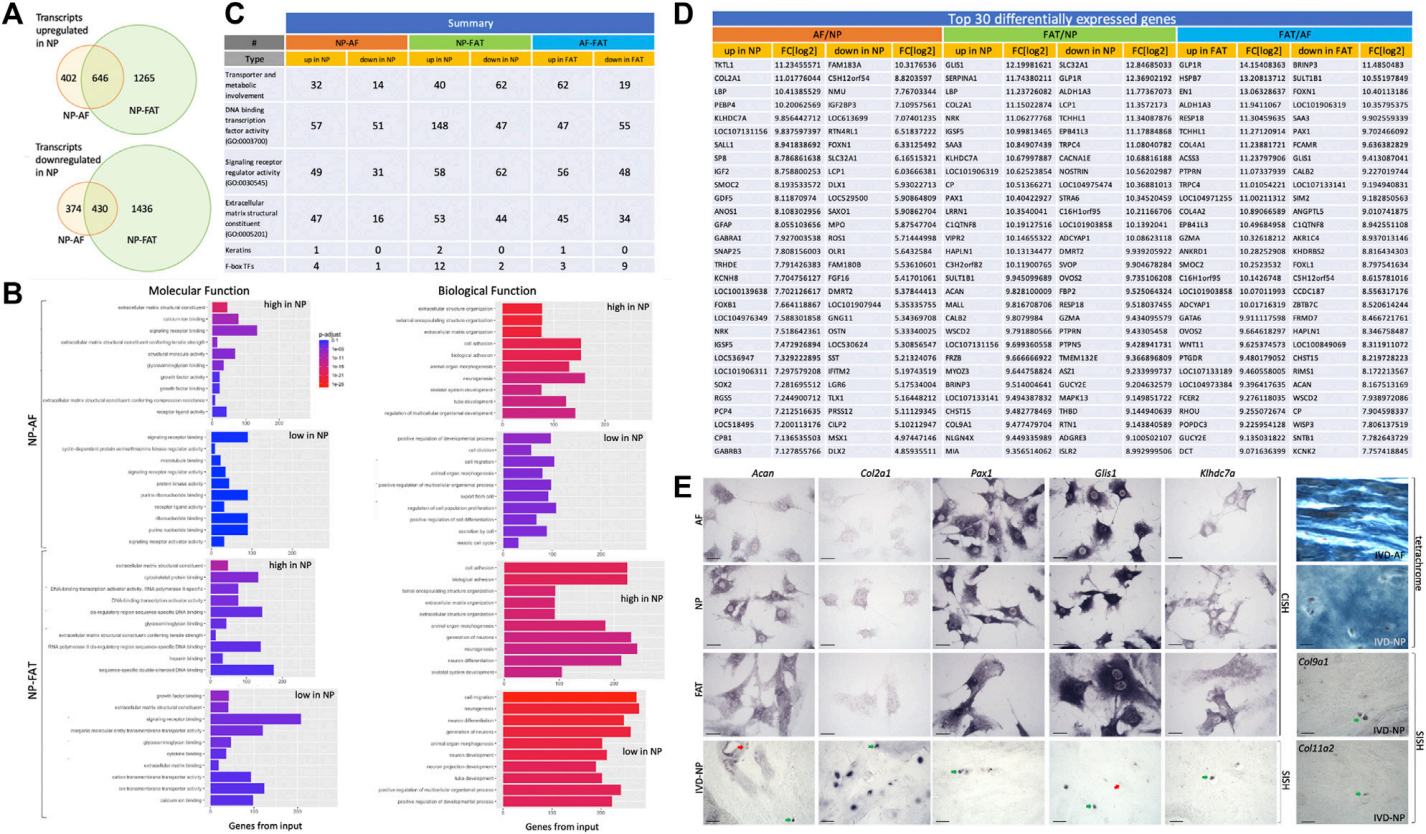
Overview of differentially expressed transcripts from annulus fibrosus (AF), nucleus pulposus (NP), and subcutaneous adipose (FAT)-derived primary cells **(A)**. Bar graphs indicate the 10 top categories after functional enrichment for molecular or biological functions. Colors indicate the Benjamini–Yekutieli adjusted *p*-values. **(B)**. Summary of RNASeq results **(C)**. Top 30 differentially expressed transcripts **(D)** and validation of selected targets through RNA *in situ* hybridization and alkaline phosphatase reporter gene expression on cultured cells (CISH) and sections through the nucleus pulposus of the IVD (SISH) where green arrows point to positive and red arrows to negative cells. Tetrachrome staining of IVD sections provides ECM histology for reference **(E)**. Scale bar represents 50 μm. Cells were isolated from a single donor to avoid the genotype effect. Cells were analyzed as three replicates for AF and NP and two replicates for FAT cells.

**FIGURE 5:**
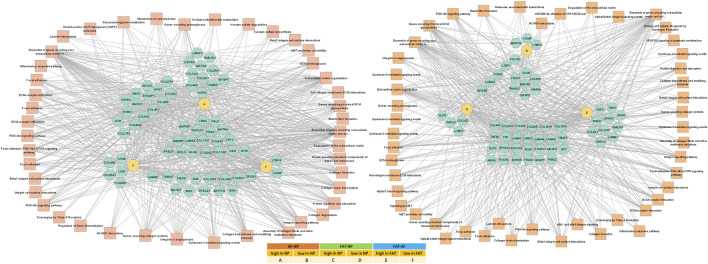
Network of differentially expressed transcripts between annulus fibrosus (AF) and nucleus pulposus (NP) cells of the IVD and adipose (FAT) stromal cells associated with the functional enrichment term extracellular matrix structural constituent (GO:0005201). Networks were generated with ToppCluster and analyzed in Cytoscape. Cells were isolated from a single donor to avoid the genotype effect. Cells were analyzed as three replicates for AF and NP and two replicates for FAT cells. Nodes are identified in the legend.

RNASeq transcriptome analysis was based on three replicates for AF and NP cells and two replicates for FAT cells. All cells were derived from the same donor to avoid genotype-related expression differences. Focusing on the NP cell phenotype, we found a nearly equal number of transcripts higher or lower in NP compared to the AF and FAT cells. More genes were differentially expressed between NP and FAT compared to NP and AF cells *in vitro* (Figure 4A). Functional enrichment analysis (Figure 4B, Supplementary Figure S2) showed that transcripts identified as higher in the NP were predominantly involved with structural components of the ECM (GO:0005201), extracellular structure organization (GO:0043062), and cell adhesion (GO:0007155). Higher transcripts in NP over FAT cells showed enrichment for DNA binding transcription activator activity (GO:0001228). Amidst the lowest transcripts in NP over FAT cells were those involved in neurogenesis (GO: 0022008) and neuron differentiation (GO:0030182). Comparing NP to AF cells, transcripts higher in NP cells were associated with the molecular function of ECM structural constituent (GO:0005201) and Ca^2+^ ion binding (GO:0005509). A numerical summary of selected transcripts is provided in Figure 4C. *Col2a1*, *Pax1*, and *Acan* were among the top 30 transcripts higher in NP over FAT cells, *Pax1* and *Acan* were in the top 30 transcripts lower in FAT over AF cells, and *Col2a1*was the second highest differentially expressed gene in NP over AF cells (Figure 4D). The top transcript higher in NP over FAT cells was *Glis1*. The lowest transcript levels in this grouping had *Slc32A1*. The highest transcript levels in FAT over AF cells were noted for *Glp1r*, and the lowest were noted for *Brinp3*. Selected transcripts were validated by RISH on cells *in vitro* and *in vivo* (Figure 4E), including *Klhdc7a*, a transcript higher in NP cells, encoding a not well-characterized Kelch-domain containing protein and two collagen transcripts, *Col9a1* and *Col11a2*, also higher in NP cells (Figure 4E, Figure 5 and Supplementary Table S1).

### 3.3 Cell-type-specific approaches toward replenishing the ECM *in vitro*

One of the top-ranking terms after functional enrichment analysis of the differentially regulated transcripts was ECM structural component (GO:0005201) (Figure 4B, Figure 5 Supplementary Table S1), with collagen as a major contributor. We found many collagen transcripts higher in NP cells upon moving into 2D culture [*n* = 15 (AF), *n* = 16 (FAT)] and fewer were lower [*n* = 2 (AF) and *n* = 9 (FAT)]. *Col2a1*, was found to be >2,000-fold higher in NP cells. *Col1a1* was reduced in NP over AF cells, and *Col1a2* expression was lower in NP versus FAT cells. A similar number of collagen transcripts was higher (*n* = 9) and lower (*n* = 7) in FAT over AF cells, with 7 of the higher transcripts identical for both IVD cell types, including two isoforms each for collagen IV and collagen V and *Col4a1* and *Col4a2* transcripts being the highest ECM related transcripts in FAT cells. *Acan* expression was 1,000x higher in NP over FAT cells and slightly elevated over AF cells, placing it in a group of transcripts higher in NP/IVD over FAT cells (Figure 5, Supplementary Table S1). Furthermore, in that group are transcripts for the large PG lubricin (Prg4) and other PG transcripts such as *Npnt*, *Matn4*, and *Fbn2*, along with transcripts for members of small leucine-rich proteoglycans (SLRPs), such as osteoglycin (Ogn) and prolargin (PRELP) (Figure 5, Supplementary Table S1). Transcripts for Abi3bp, a protein with glycosaminoglycan (GAG) binding affinity, and transcripts for the proteoglycan-link protein Hapln1 are considerable, and those for the SLRPs biglycan (Bgn) and fibromodulin (Fmod) were slightly lower in FAT cells. Other transcripts with a fold-change of ∼10 or higher in NP over FAT cells included *Anos1* and *Bmper*. *Vwa5a* transcripts were higher in FAT and down in NP cells.

### 3.4 Transcription and signaling factor networks in early 2D culture

**FIGURE 6:**
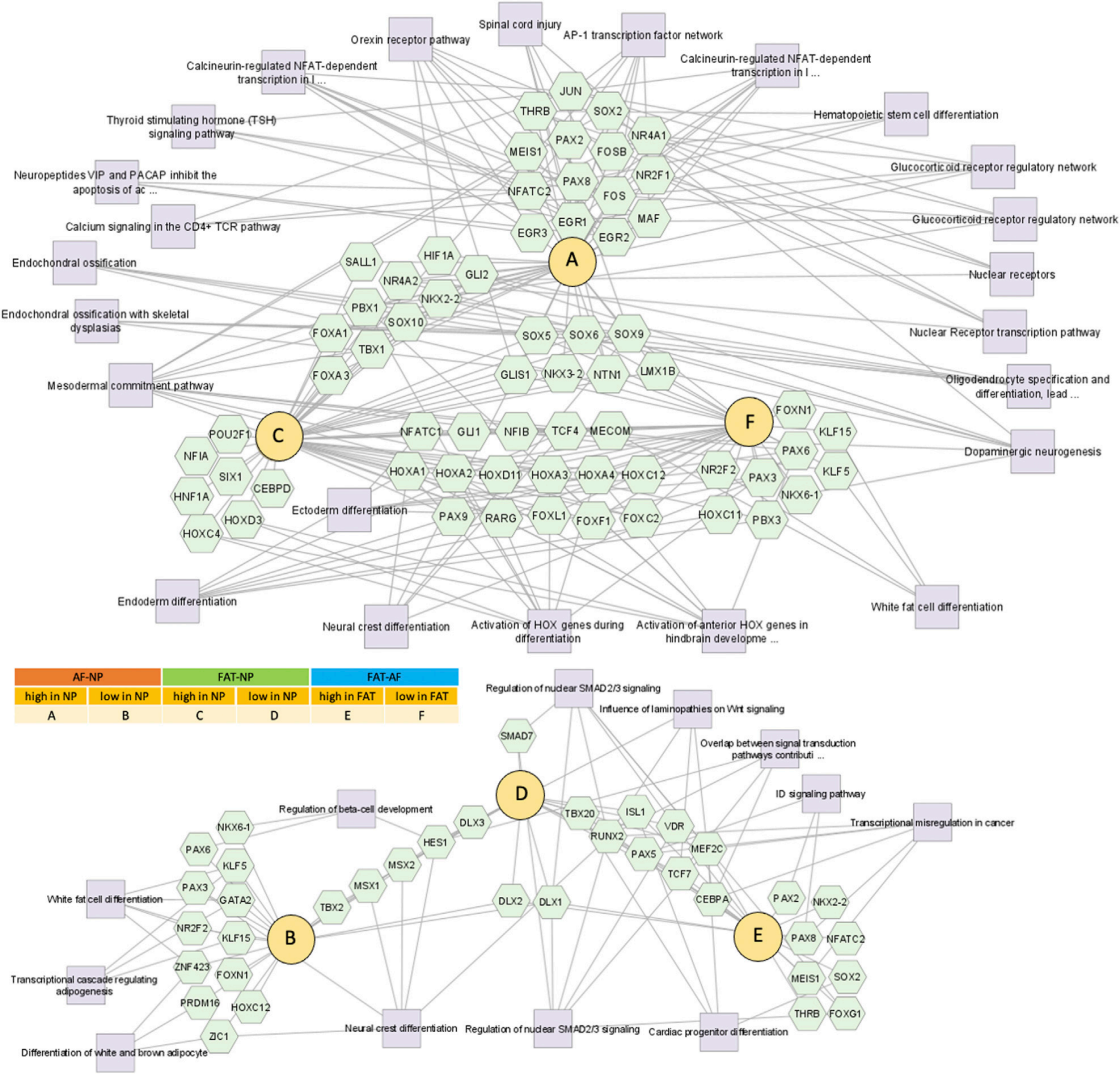
Network of differentially expressed transcripts between annulus fibrosus (AF) and nucleus pulposus (NP) cells of the IVD and adipose (FAT) stromal cells associated with the functional enrichment term DNA binding transcription factor activity (GO:0003700). Networks were generated with ToppCluster and analyzed in Cytoscape. Cells were isolated from a single donor to avoid the genotype effect. Cells were analyzed as three replicates for AF and NP and two replicates for FAT cells. Nodes are identified in the legend.

**FIGURE 7:**
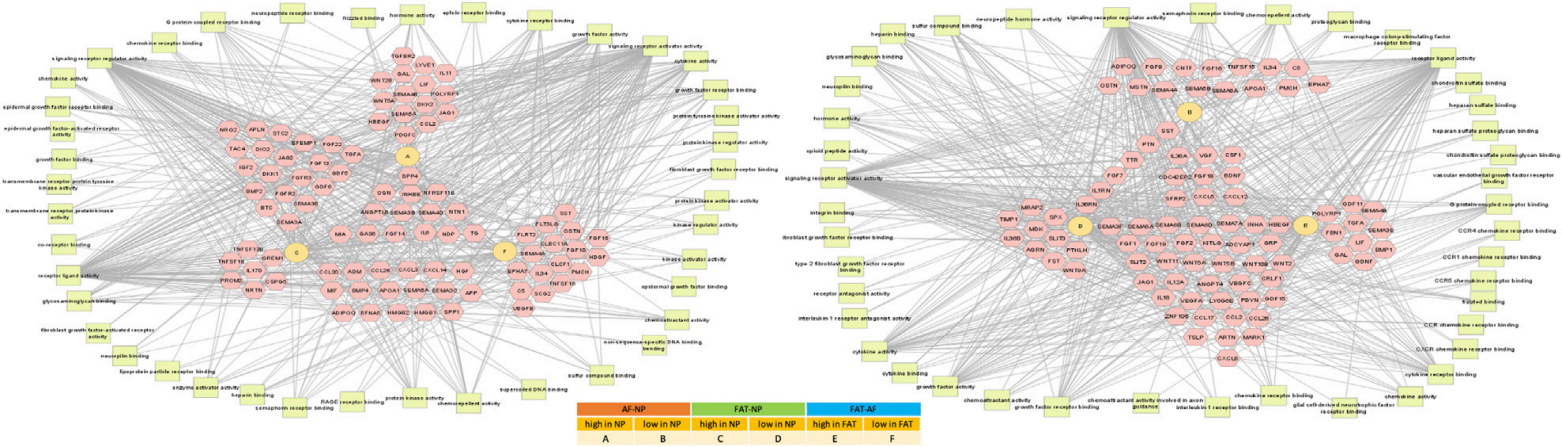
Network of differentially expressed transcripts between annulus fibrosus (AF) and nucleus pulposus (NP) cells of the IVD and adipose (FAT) stromal cells associated with the functional enrichment term signaling receptor regulator activity (GO:0030545). Networks were generated with ToppCluster and analyzed in Cytoscape. Cells were isolated from a single donor to avoid the genotype effect. Cells were analyzed as three replicates for AF and NP and two replicates for FAT cells. Nodes are identified in the legend.

Pathway association-based gene regulatory trends were analyzed between IVD (NP, AF) and subcutaneous FAT-derived primary cells (Figure 6, Supplementary Table S2). *Pparg* was over 50x elevated in FAT over IVD cells. Transcripts higher in NP/IVD over FAT cells included *Sox5*, *Sox6*, *Sox9*, *Bapx1* (*Nkx3.2*), *Lmx1b*, and *Ntn1*. *Glis1* showed the highest transcript level in NP over FAT cells. *Gli1* was higher in IVD over FAT cells and *Gli2* in NP cells (Figure 6, Supplementary Table S2). *Tbx1* (*Tbox*) transcripts were higher in NP than AF (10x) and FAT (239x) cells. We also found transcripts of six members of the Hox A, C, and D cluster higher in NP over FAT cells along with *Pax9*, involved in IVD patterning (Peters et al., 1999; Sivakamasundari and Lufkin, 2012; Sivakamasundari et al., 2017). *Dlx1*/*Dlx2* transcripts were lower in NP/IVD over FAT cells and *Msx1*/*Msx2* transcripts in NP over AF and FAT cells. *Runx2* transcript levels were higher in FAT over IVD-derived cells, with a more than fivefold increase compared to NP cells (Supplementary Table S2). *Sox10* transcripts were higher in NP cells. *Pax3* and *Pax6* transcripts were generally elevated in AF cells along with *Foxn1* transcripts. *Pax9* expression was lower in FAT cells (Figure 6, Supplementary Table S2). Furthermore, higher transcript levels in IVD over FAT cells were found for the semaphorins *Sema3b* and *Sema4d*, along with *Mia* and *Gas6* transcripts. *Tnfsr11b* transcript levels were up to 478x higher in IVD over FAT cells. Another group of transcripts was lower in NP/IVD over FAT cells, with some of the highest fold changes observed for *Sfrp2*, *Vgf*, *Fgf18*, and *Cxcl3* (Figure 7, Supplementary Table S3).

### 3.5 Transcripts involved in cytokine responses and ECM remodeling

The highest number of differentially regulated transcripts was found between NP and FAT cells (Supplementary Table S4. Transcript levels for Il12A, Il18 and its receptor, and Il21R, as well as Il1RL1, were increased in FAT cells. Expression of stromal cell-derived factor 1 (*Cxcl12*) was reduced in NP over FAT cells. Transcripts encoding the cytokines Il6, Cxcl14, and Cxcr4 were increased in IVD over FAT cells (Supplementary Table S4). Differentially expressed transcripts for metalloproteinases, such as matrix metalloproteinase (Mmp), disintegrin, and metalloproteinases (Adam), and those with thrombospondin motifs (Adamts) higher in IVD/NP over FAT cells included *Adamdec1*, *Adam23*, *Mmp3*, and *Mmp13*. More transcripts were higher in FAT cells such as *Adamst14*, *Mmp9*, *Mmp12*, and *Mmp23b* (Supplementary Table S5).

### 3.6 Differential gene expression suggests metabolic adaptions in NP stromal cells

**FIGURE 8:**
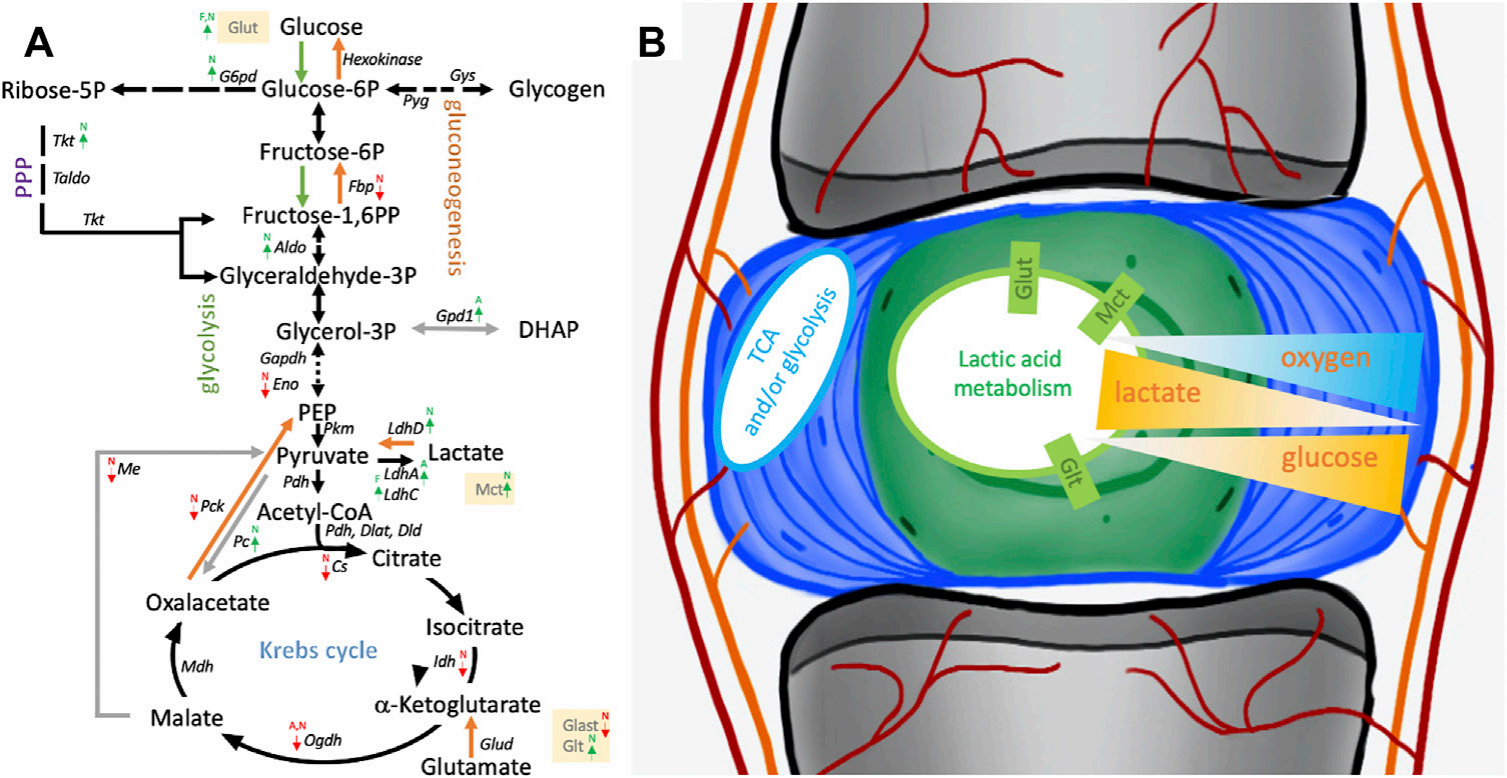
Schematic of pathways involving glucose and lactate metabolism. **(A)** Arrows indicate differentially expressed transcripts encoding for an enzyme or transporter in cells derived from the nucleus pulposus (N), annulus fibrosus (A), or adipose (F) tissue. **(B)** Illustrates NP niche conditions, metabolic pathways and gradients of crucial contributors to the NP niche condition alongside their transporters. Cells were isolated from a single donor to avoid the genotype effect. Cells were analyzed as three replicates for AF and NP and two replicates for FAT cells. P, phosphate; PPP, pentose phosphate pathway; TCA, tricarboxylic acid cycle.

An up to a 5x increase in *Slc2a1* transcripts encoding Glut1 was noted in NP over AF cells along with *Slc2a3*, *Slc2a10*, and *Slc2a11* encoding further glucose transporters. FAT cells showed an up to 15x increase in *Slc2a3* transcript levels over IVD cells (Figure 8; Table 3). An over 2x increase in *G6pd* expression in NP over AF cells was noted. *Tktl1* transcripts were highest comparing NP to AF cells (2,400x) and increased over FAT cells (200x) (Figure 8A; Table 3). Furthermore, higher transcripts levels were noted in NP over FAT encoding two fructose-1,6-bisphosphate (F1,6PP) aldolase isoforms AldoA (2x) and AldoB (>4x). Transcripts of *Eno2* encoding for an enolase isoform involved in generating phosphoenol-pyruvate (PEP) were lower in NP cells over AF and FAT cells (Table 3). Pyruvate kinase (*Pkm*) transcript levels were not significantly different. Transcripts for different isoforms of lactate dehydrogenase (*Ldh*) were differentially expressed in the three cell types (Figure 8A; Table 3). *LdhA* transcripts doubled in AF over FAT cells, whereas *LdhC* transcription was doubled in FAT over AF cells. NP cells showed an up to 18x increase in *LdhD* transcripts. Monocarboxylic transporter proteins (Mct) are encoded by the *Slc16* gene family. Expression of *Slc16a2* (Mct8) and *Slc16a3* (Mct4) was increased >4x in NP over AF cells, and *Slc16a2, Slc16a3*, *Slc16a6* (Mct6), and *Slc16*a7 (Mct2) transcript levels were higher in NP over FAT cells, with *Slc16a2* transcripts being increased by 247x (Figure 8; Table 3). Transcripts for the gluconeogenesis associated fructo-bi-phosphatase 2 (Fbp2) were over 700x lower in NP versus FAT cells (Figure 8A; Table 3). A threefold increase in transcripts encoding pyruvate carboxylase (Pc) was seen in NP over FAT cells. However, transcripts for many enzymes converting the various metabolites of the Krebs cycle were lower in NP cells (Figure 8A; Table 3). Transcripts for the mitochondrial phosphoenolpyruvate carboxykinase 2 (Pck2) were reduced in NP over AF and/or FAT cells (Table 3). *Me3* transcripts encoding a pyruvic-malic carboxylase (malic enzyme) were also lower in NP cells, and an up to 40x reduction of transcripts for the glutamate aspartate transporter 1 (Glast1) encoded by *Slc1a3* was noted in NP cells. In contrast, transcript levels for ATP-citrate lyase (Acly) and malate dehydrogenase (Mdh) were not significantly different (Figure 8; Table 3). Transcripts for glutamate transporters (Glt) were higher in NP over FAT cells [*Slc1a1* (5x), *Slc1a2* (10x)]. Several other Glt transcripts were up to 17x higher in FAT over NP cells (Table 3). Transcripts for glutamate dehydrogenase (*Glutd*) were not increased in NP cells, nor were those for oxoglutarate dehydrogenase (*Ogdh*).

**TABLE 3:** List of differentially expressed genes between annulus fibrosus (AF) and nucleus pulposus (NP) cells of the IVD and adipose (FAT) stromal cells of the same donor associated with transporter or enzymatic function as it is related to glucose and lactate metabolism. Differences in transcript levels are displayed as log_2_ fold changes (FC[log_2_]).

Transporter and metabolic involvement											
NP-AF				NP-FAT				AF-FAT			
High in NP	FC[log_2_]	Low in NP	FC[log_2_]	High in NP	FC[log_2_]	Low in NP	FC[log_2_]	High in FAT	FC[log_2_]	Low in FAT	FC[log_2_]
ABCA1	1.107416	ABCA13	3.0423657	ABCA2	2.3704272	ABCA10	5.37703174	ABCA1	1.6603812	ABCA13	6.9860053
ABCA7	1.8495978	ABCB1	3.71542703	ABCA7	2.9035689	ABCA6	5.00974356	ABCA10	4.86389688	ABCA2	1.8733881
ABCA9	1.217001	ABCC6	2.0047123	SLC13A4	5.3811892	ABCB1	4.54434519	ABCA6	4.75565046	ABCA7	1.0499226
SLC12A5	1.4976994	SLC17A1	1.66125258	SLC15A1	1.5187822	ABCB10	1.0057657	ABCA9	1.15909967	SLC13A4	5.0996368
SLC16A2	2.3809349	SLC1A3	3.92477973	SLC16A2	2.9604195	ABCC9	6.06613899	ABCC9	5.61257556	SLC15A1	1.9223883
SLC16A3	2.0853338	SLC32A1	6.16515321	SLC16A3	7.9458964	ABCG2	1.8490283	ABCG2	1.06999128	SLC16A3	5.8578588
SLC17A9	1.0910986	SLC38A5	2.66219258	SLC16A7	1.1447606	SLC11A1	3.67694045	SLC10A7	1.06335743	SLC16A7	1.6472511
SLC1A1	2.4249755	SLC4A8	1.85662582	SLC16A9	3.2773663	SLC11A2	1.00803354	SLC11A1	2.31949877	SLC1A2	3.1922495
SLC26A2	1.1107333	SLC5A10	2.73574138	SLC1A1	1.8362134	SLC12A5	1.36857322	SLC12A5	2.85884147	SLC27A3	1.5234197
SLC2A1	1.1836085	SLCO4A1	2.79114098	SLC1A2	3.1819722	SLC12A7	2.03733657	SLC12A7	1.86292524	SLC27A5	2.3560138
SLC2A10	1.2492746	MKI67	1.01587988	SLC24A3	1.344162	SLC13A3	4.35570689	SLC13A3	4.11526361	SLC2A4RG	1.6076744
SLC2A11	2.2913235	ADH6	1.70036695	SLC25A1	1.0998811	SLC13A5	4.89617615	SLC13A5	3.59392955	SLC37A1	3.8230037
SLC2A3	1.174152	ENO2	1.62413621	SLC25A37	1.1640673	SLC16A10	1.78139877	SLC16A14	3.53114418	SLC39A12	2.5068281
SLC34A3	1.9024991	PCK2	1.32290575	SLC26A2	1.280204	SLC16A11	1.53543731	SLC16A5	1.28748552	SLC46A3	1.802791
SLC37A2	2.0300991			SLC27A3	1.1717116	SLC16A14	3.46538338	SLC18A1	1.95600621	SLC4A8	1.2705636
SLC38A1	1.0515642			SLC27A5	3.0629731	SLC16A5	1.7872539	SLC19A1	1.02173277	SLC9A3R2	1.612095
SLC38A3	4.2240492			SLC28A1	1.2366143	SLC18A1	1.66337459	SLC1A3	1.32367679	SLC9C2	1.7532298
SLC38A4	1.9272223			SLC29A1	1.0512012	SLC19A1	1.10445905	SLC1A4	1.50720805	ADH6	1.0187314
SLC39A12	1.4951142			SLC2A4RG	1.4397429	SLC1A3	5.24874541	SLC1A7	2.74889464	ENO3	1.66983792
SLC4A10	1.1422997			SLC34A3	2.3165404	SLC1A4	1.82289712	SLC22A15	1.69960741	GPD1	1.0187314
SLC4A11	1.5194752			SLC35F3	4.0935286	SLC1A7	4.0383087	SLC22A23	1.60894006	LDHA	1.06531238
SLC6A12	1.7317152			SLC37A1	3.9888562	SLC20A2	1.14204345	SLC23A2	1.98319545		
SLC6A16	2.4543627			SLC37A2	2.7910528	SLC22A15	2.56326155	SLC25A40	1.84301676		
SLC6A6	1.9270614			SLC38A3	3.6816731	SLC22A4	1.42381369	SLC29A2	1.0783102		
SLC7A4	2.197844			SLC38A4	1.9919599	SLC23A1	1.92323893	SLC29A4	1.81277161		
SLC7A8	2.1103889			SLC39A12	4.0091462	SLC23A2	1.57282683	SLC2A10	1.4818014		
SLC8A3	1.7427815			SLC44A1	1.3703218	SLC25A13	1.07551127	SLC2A11	2.53101093		
SLC9A3	1.9028267			SLC46A3	2.2629187	SLC25A34	1.11133798	SLC2A13	1.67203706		
SLC9A3R2	1.2496462			SLC47A1	1.4676526	SLC25A40	2.25337881	SLC2A3	3.96381542		
SLC9A7	4.366765			SLC4A10	1.8307096	SLC2A3	2.79262243	SLC30A1	1.44596986		
G6PD	1.1847245			SLC5A1	6.3519744	SLC30A1	1.31422323	SLC30A3	1.87263311		
LDHD	2.6162837			SLC6A12	4.7358634	SLC30A3	3.63017143	SLC32A1	6.67564447		
				SLC7A4	2.0816688	SLC31A1	1.02772407	SLC35B4	1.02592009		
				SLC7A8	1.6367815	SLC31A2	1.20213609	SLC35G2	2.45473731		
				SLC9A3R1	1.1517147	SLC32A1	12.8468503	SLC36A1	1.23811746		
				SLC9A3R2	2.8587365	SLC33A1	1.11548123	SLC38A5	1.1088024		
				SLC9A7	6.6786746	SLC35F6	1.02748112	SLC39A4	2.46108567		
				ALDOB	1.0990772	SLC35G2	3.11029394	SLC43A2	1.33502719		
				ALDOC	2.2110627	SLC36A1	1.40222623	SLC43A3	1.09142882		
				LDHD	4.1594281	SLC38A5	3.77198604	SLC45A3	2.55236965		
				PC	1.632737	SLC39A4	3.07494017	SLC4A2	1.67687556		
						SLC43A3	1.79495664	SLC4A3	3.57562977		
						SLC45A3	2.34494537	SLC4A4	3.14111367		
						SLC4A2	1.51796846	SLC6A16	1.6899226		
						SLC4A3	3.16939849	SLC6A17	2.50815521		
						SLC4A4	3.73956579	SLC6A2	2.28402999		
						SLC5A10	2.92049826	SLC6A20	2.55564708		
						SLC6A17	3.49143045	SLC6A6	1.11352083		
						SLC6A2	2.44988874	SLC6A7	1.86133382		
						SLC6A20	2.15540887	SLC7A5	1.17106655		
						SLC7A5	1.47287737	SLC7A6	1.62703556		
						SLC7A6	1.61257805	SLC8A1	2.13876095		
						SLC7A7	1.06729759	SLC8B1	1.41021258		
						SLC8A1	5.56692634	SLC9A1	1.14095153		
						SLC8B1	1.30473401	SLC9A3	2.14101396		
						SLCO4A1	4.64488219	SLCO4A1	1.85151118		
						SLCO5A1	2.63970373	SLCO5A1	3.635766		
						CS	1.23529324	ENO2	1.6273096		
						ENO2	3.25375867	FBP2	4.20474721		
						FBP2	9.52506432	G6PD	1.21398008		
						IDH3A	1.0098097	GPD2	1.08031657		
						ME3	1.2938424	LDHC	1.1462041		
						OGDHL	1.46559197	OGDHL	2.0675304		

### 3.7 Stromal cell proliferation, survival, and tumorigenic potential

We did not see a significant difference in *Oct4* transcripts in 2D culture. However, transcript levels for its heterodimer partner Sox2 were over 128x higher in NP and FAT over AF cells. We also noted an increase in *Lif* expression in NP and FAT over AF cells and increased transcripts for the Lif receptor in NP over FAT cells (Table 5, Supplementary Table S2). Transcripts for Sox2/Oct4 downstream targets, such as *Klf4 and Top2a*, were slightly higher in NP over FAT cells, whereas *Mras* was slightly higher in FAT over IVD cells (data not shown). Transcript levels of *Glis1* were high in IVD, especially NP over FAT cells (Figure 4D, Supplementary Table S2) when maintained in 2D culture with standard media. This was supported by CISH results (Figure 4E). *Glis1* was expressed in a subpopulation of NP cells in sections of the IVD, as shown by SISH (Figure 4E, green arrow).

**FIGURE 9:**
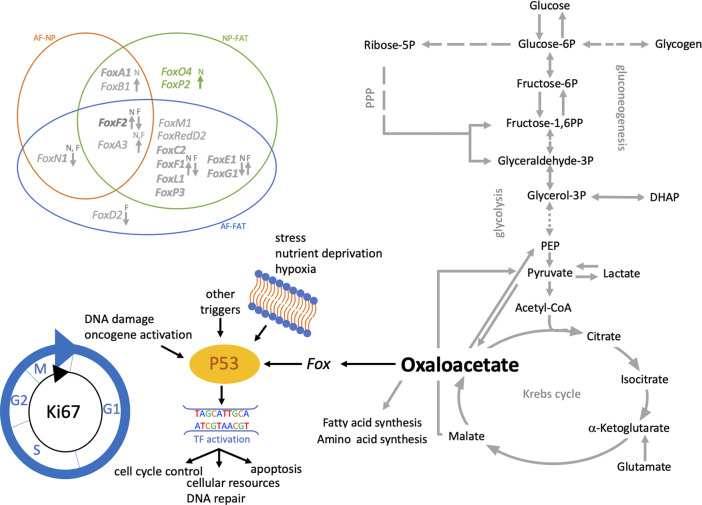
Schematic of pathways involving glucose and lactate metabolism leading to oxalacetate production, which modulates cell function through Fox transcription factors. Venn diagram shows the differentially expressed *Fox* genes between IVD (AF, NP) and adipose (FAT) derived cells. Those with a fold change of 3 or higher in NP over FAT. Arrows indicate up- or downregulation. The color scheme follows Table 3. Cells were isolated from a single donor to avoid the genotype effect. Cells were analyzed as three replicates for AF and NP and two replicates for FAT cells.

Twelve Fox-TF had higher transcript levels in NP over FAT cells, with *FoxO4* levels doubled, alongside the transcript *Prkaa2.* No significant difference was seen for *FoxO4* transcript levels between NP and AF cells (Figure 3D; Table 4). *Fam183A* was lowest in NP compared to AF cells (Figure 3D; Table 4). *FoxF2* and *FoxA3* transcription levels were higher in NP cells (Figure 9; Table 5). *FoxP2* and *FoxC2* were higher in NP over FAT cells, and *FoxL*1 was the highest *Fox* transcript in IVD cells, with a 410-fold increase over FAT cells. In this context, we found that *P53* and *P73* transcripts at least doubled in IVD over FAT cells (Table 5). *P21* was not differentially expressed between the cell lines in 2D. A positive correlation between *P53*, *P73*, and *Ki67* transcript levels was noted in all cell types, with higher levels in IVD over FAT cells, whereas P53-induced protein 11(*TP53I11* or *Pig11*) showed a negative correlation and was lower in IVD over FAT cells. *Perp* and *Ros1* expression were also lower in NP over FAT cells in 2D culture (Table 5).

**TABLE 4:** Differentially expressed Fox transcription factors between annulus fibrosus (AF) and nucleus pulposus (NP) cells of the IVD and adipose (FAT) stromal cells of the same donor displayed by the log_2_ fold changes (FC[log_2_].

Fox transcription factor signaling											
NP-AF				NP-FAT				AF-FAT			
High in NP	FC[log_2_]	Low in NP	FC[log_2_]	High in NP	FC[log_2_]	Low in NP	FC[log_2_]	High in FAT	FC[log_2_]	Low in FAT	FC[log_2_]
FOXA1	2.6017173	FOXN1	6.33125492	FOXA1	2.8652228	FOXE1	8.21663533	FOXA3	1.2072111	FOXC2	4.941075
FOXA3	2.2159301			FOXA3	1.0101592	FOXG1	1.92838748	FOXE1	8.14658753	FOXD2	1.2029102
FOXB1	7.6641189			FOXB1	0.0922735			FOXG1	3.00153231	FOXF1	3.9939037
FOXF2	1.120343			FOXC2	5.2344779					FOXF2	3.1880346
				FOXF1	4.1897013					FOXL1	8.7975416
				FOXF2	4.3065908					FOXM1	2.249502
				FOXL1	8.6824443					FOXN1	10.401132
				FOXM1	1.4568767					FOXP3	1.0594869
				FOXO4	1.1305628					FOXRED2	1.0279899
				FOXP2	1.1101717						
				FOXP3	1.6949361						
				FOXRED2	1.0900497						

**TABLE 5:** Differentially expressed genes related to cell proliferation and differentiation between annulus fibrosus (AF) and nucleus pulposus (NP) cells of the IVD and adipose (FAT) stromal cells of the same donor displayed by the log_2_ fold changes (FC[log_2_]).

Cluster of differentiation, proliferation, and tumor markers											
AF-NP				FAT-NP				FAT-AF			
High in NP	FC[log_2_]	Low in NP	FC[log_2_]	High in NP	FC[log_2_]	Low in NP	FC[log_2_]	High in FAT	FC[log_2_]	Low in FAT	FC[log_2_]
CD101	1.1087351	CD200	3.08514334	CD101	3.5796512	CD1D	2.55423585	CD1D	5.36243721	CD101	2.4821292
CD1D	2.811693	CD248	2.17560969	CD109	1.1754791	CD200	5.15483736	CD200	2.067109	CD247	2.590695
Thy1	1.684195	CD34	2.3454104	CD247	2.1609358	CD248	6.28247479	CD248	4.10536467	CD302	1.1689161
WWP2	1.4974154	CD3G	2.41875435	CD302	1.0143442	CD274	1.378496	CD274	1.21713399	CD55	4.1310761
LIF	3.6679379	CD55	1.46401601	CD55	2.6657553	CD300A	8.42767478	CD300A	5.91131024	CD83	2.2819783
LIFR	1.1041282	CD83	1.49757962	Thy1	2.063586	CD34	3.30971672	CD36	2.49720948	MKI67	2.2445026
		MKI67	1.01587988	MKI67	1.2254082	CD36	2.93511372	CD37	1.39069521	TP53	1.087811
		TP73	1.60769146	PRKAA2	1.536482	CD37	1.63810686	CD68	1.64888685	TP73	3.4991516
				TP53	1.2037639	CD3G	2.80666721	CD82	1.24993244	WWP2	1.3393384
				TP73	1.8892565	CD40	1.31565778	ALDH1A1	8.21548452		
				WWP2	2.8340407	CD59	1.24383598	ALDH1A3	11.9411067		
				GLIS1	12.199816	CD68	1.17167402	ALDH4A1	1.08792535		
				LIFR	1.0732595	CD82	1.06014684	ALDH8A1	5.97006324		
				CAV	1.0615384	ALDH1A1	7.98238535	TP53I11	7.52357485		
						ALDH1A3	11.7736707	LIF	3.44013072		
						ALDH1L2	1.13162783				
						ALDH4A1	1.31313698				
						ALDH8A1	7.05690981				
						PERP	2.21093747				
						ROS1	6.09888093				
						TP53I11	5.68842397				

Several clusters of differentiation (CD) marker transcripts were higher in IVD over FAT-derived cells (Table 5). *CD90* transcripts were fourfold higher in NP over FAT cells and twofold increased over AF cells. Transcripts encoding CD101 were higher in NP cells compared to AF and FAT cells (Table 5). *CD109* transcript levels were higher in NP over FAT cells. Transcript levels for CD36, CD200, CD248, and CD300 were reduced in NP and/or IVD over FAT cells (Table 5). Lastly, aldehyde dehydrogenase (Aldh) transcripts encoding isoforms of Aldh1a1, Aldh1a3, and Aldh8a1 were ∼60–4,000x increased in FAT over IVD cells (Table 5).

### 3.8 Performance of NP and FAT-derived stromal cells in 3D culture

**FIGURE 10:**
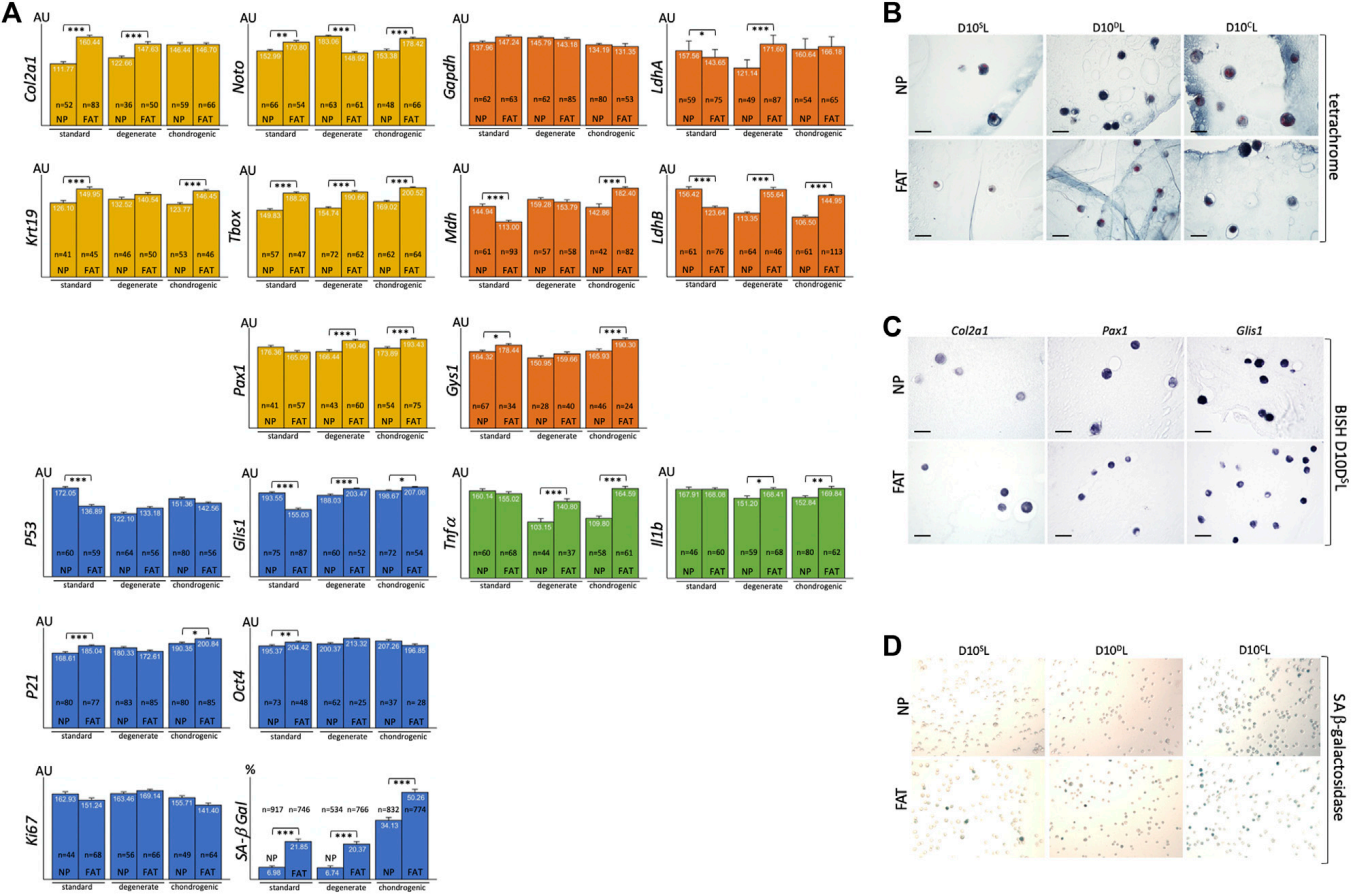
Bar graphs indicate RNA levels after BISH and ImageJ analysis of NP and FAT cells after 10 days of 3D alginate bead culture under different culture conditions for genes related to the nucleus pulposus (NP) (yellow), glucose and lactate metabolism (orange), inflammatory markers (green), cell proliferation and senescence markers, and SA-beta galactosidase staining (blue). Cells were isolated from a single donor to avoid genotype effects. A minimum of 25 cells were analyzed after subjecting three beads per probe, cell line, and condition to BISH **(A)**. Tetrachrome-based histology of 7 μm paraffin sections of NP and FAT cells in alginate beads after 10 days of 3D alginate bead culture under different culture conditions shows the round cell morphology **(B)**. Examples of 7 μm paraffin sections of NP and FAT cells after 10 days of 3D alginate bead culture under different culture conditions and BISH show AP-reporter gene activity for *Col2a1*, *Pax1*, and *Glis1* expression **(C)**. Example of images used for a cell count of NP and FAT cells after 10 days of 3D alginate bead culture under different culture conditions and subjection to SA-β-galactosidase stain **(D)**. AU, arbitrary units; S, standard media D10^S^L. D, Media D10^D^L mimicking degenerate conditions. C, chondrogenic D10^C^L media. Significance is indicated as *** (*p* < 0.001); **(*p* < 0.01); *(*p* < 0.05); *n* indicates the number of individual cells analyzed for BISH. Scale bar represents 50 μm.

NP and FAT cells in 3D culture were exposed to standard media (D10^S^L), degenerate conditions mimicking the IVD niche pH (D10^D^L), and chondrogenic conditions (D10^C^L) through supplements added to the D10^S^L media as described and subjected to BISH gene expression analysis. Cells were isolated from the same donor animal to avoid genotype-related expression differences. Three beads were used as technical replicates, and a minimum of 25 cells were analyzed for each gene, cell type, and condition (Figure 10A).

#### 3.8.1 Extracellular alginate scaffold influences cell phenotype

Using 1.2% alginate does not promote AF cell morphology *in vitro* (Figure 3E). The latter were therefore excluded from this comparison. A morphological change from flat, spread-out, to round was noted upon moving NP and FAT cells into the 3D alginate-based environment (Figure 4E, Figure 10B). In 3D culture using standard D10^S^L media, *Col2a1* and *Krt19* expression along with TFs such as *T-box* and *Noto* was higher in FAT over NP cells (Figure 10A). Transcripts for the metabolic enzyme Gys1 were also elevated in FAT over NP cells (Figure 10A). Transcript levels for *LdhA*, particularly *LdhB* and *Mdh*, were higher in NP over FAT cells (Figure 8, Figure 9, Figure 10A). Expression of *Glis1* remained higher in NP over FAT cells, but *Oct4* levels were now increased in FAT cells (Figure 4E, Figures 10A,C). *P53* levels remained higher, and *P21* levels were lower in NP cells. A higher proportion of senescent cells was noted in the FAT cell lineage in 3D culture as identified by SA β-galactosidase staining (Figures 10A,D). Transcript levels for the inflammatory markers Tnfα and Il1b were not significantly different between the two cell lines in 3D under standard conditions (Figure 10A).

#### 3.8.2 Adaption to partial degenerate NP niche conditions

To mimic some attributes of the degenerate NP, lactic acid was added to the culture medium for pH adjustment to pH6.5 (D10^D^L). Expression of *Col2a1*, *T-box*, and *Pax1* was significantly higher in FAT cells, whereas *Noto* was now higher in NP cells. Resulting from a significant increase in FAT *Pax1*, yet a decrease in *Noto* expression between standard and degenerate conditions and an increase in NP *Noto* (Figure 10A, Supplementary Figure S1). Upon changing from standard to degenerate media, no significant changes in *Glis1*, *Oct4*, or *Ki67* levels were noted in NP cells, whereas an increase was noted for all three transcripts in FAT cells, raising *Glis1* and *Oct4* expression in FAT cells above those in NP cells. The proportion of senescent cells remained significantly higher in FAT cells with no significant difference in *P53*, *P21*, or *Ki67* transcripts between the cell lines. However, the addition of lactic acid reduced *P53* and increased P21 expression in NP cells, whereas *P21* levels decreased and *Ki67* levels increased in FAT cells (Figures 10A,D, Supplementary Figure S1). For glucose/lactate metabolism-related enzymes, a significant increase for *LdhA/B* was noted in FAT over NP cells due to reduced expression in NP and an increase thereof in FAT cells when changing from standard to degenerate medium (Figure 10A, Supplementary Figure S1). Expression of *Tnfa* decreased significantly in NP cells between normal and degenerate conditions, with both inflammatory markers (*Tnfα, Il1b*) being significantly higher in FAT over NP cells (Figure 10A, Supplementary Figure S1).

#### 3.8.3 Adaption to partial chondrogenic conditions

Although *Col2a1* transcript levels were similar between NP and FAT cells in chondrogenic (D10^C^L) conditions, they were at their highest for NP cells (Supplementary Figure S1). *Krt19*, *Noto*, *T-box*, and *Pax1* expression was increased in FAT over NP cells, and the three TFs were at their highest level in FAT cells (Figure 10A, Supplementary Figure S1). From a metabolic perspective, transcript levels for *Gys1*, *Mdh*, and *LdhB* were significantly lower in NP over FAT cells (Figure 10A), and *Mdh* and *LdhA/B* transcripts were significantly increased in the FAT lineage between standard and chondrogenic conditions, whereas *LdhB* decreased in the NP lineage (Supplementary Figure S1). The proportion of senescent cells remained higher in the FAT lineage. However, for both cell lines, the highest proportion of senescent cells and *P21* transcript levels was noted under chondrogenic conditions (Figure 10A, Supplementary Figure S1). *Glis1* expression was slightly higher in FAT over NP cells (Figure 10A). Transcript levels for the inflammatory markers *Tnfα* and *IL1b* were significantly higher in FAT over NP cells under chondrogenic conditions.

## 4 Discussion

Regenerative medicine employs cell-based therapies amidst other strategies (Kraus et al., 2021). Stem or progenitor cells are a valuable but sparse resource and typically require expansion in culture for quality control and enrichment purposes prior to therapeutic applications. For practical reasons, this expansion is most feasible in 2D monolayer culture, an often-scrutinized approach, as it represents an unnatural environment for cells removed from complex ECM-rich tissues. The use of adult mesenchymal stem or stromal cells is appealing, especially if environmental cues can be used efficiently for somatic cell reprogramming and differentiation. However, these processes occur in a lineage-specific manner, and more insight into the complicated, interrelated lineage relationship of adult stem cells is needed because their ability to differentiate into a particular cell lineage and long-term survival in an aging tissue are important (Zhou et al., 2019). Settling into an existing tissue niche challenges transplanted cells, especially if the niche is not a safe harbor.

### 4.1 Stromal cell energy sources

Serum, typically FBS, delivers growth factors, some glucose, and other undefined components to promote life *in vitro*. Compositions vary between suppliers and production lots and can impact experimental outcomes (Kraus et al., 2018). A connection between growth factors, their effect on glucose metabolism, and cell proliferation exists (Vander Heiden et al., 2001). Glucose is an energy source for cells *in vivo* and *in vitro* under aerobic and anaerobic (lactic acid fermentation) conditions and serves as a building block for macromolecules; hence, the culture medium is typically enriched with glucose. However, the NP of a healthy mature IVD is avascular, with few cells residing in an abundance of ECM under low-nutrient conditions where lactic acid production lowers the pH (Diamant et al., 1968; Nachemson, 1969; Urban et al., 1977; Razaq et al., 2000; Razaq et al., 2003; Urban and Roberts, 2003; Bibby and Urban, 2004; Urban et al., 2004; Bibby et al., 2005; Wuertz et al., 2008; Wuertz et al., 2009; Gilbert et al., 2016). For survival, NP cells likely adapted their metabolism to these conditions. Previous findings in a different context showed that constant glucose availability and lactate removal alongside a likely oversupply of growth factors through routine medium changes does not reflect the *in vivo* situation of cells, especially not those of NP cells (Cantor et al., 2017). Nutrient concentrations of commonly used media often differ from those in human plasma (Cantor et al., 2017) and are frequently high in glucose (4.5 g/L), yet many stem or stromal cells, including those of the NP, perform better under low glucose conditions (Saki et al., 2013; Liu et al., 2020; Pang et al., 2020). Low glucose medium (1 g/L, 5.5 mM), including the glucose contribution from 10% FBS, is not yet considered pre-diabetic (10 mM) (Johnson et al., 2008; Wuertz et al., 2008; Turner et al., 2016; Lyu, 2022). In our limited and simplistic 5-day culture experiment with daily medium changes, NP cells could thrive on only the small amount of glucose provided by the serum, confirming studies by others (Wuertz et al., 2008). All cell lines tolerated a reduction to 5% FBS at low glucose concentrations (1 g/L); however, only a total cell count and no live/dead assay was performed. Reduced proliferation below 5% FBS at 1 g/L glucose is likely attributable to the reduced growth factors.

Observations in rat cells suggested higher proliferation of IVD cells than adipose stromal cells in 2D (Han et al., 2014). Here, using RNASeq transcriptome profiling on bovine cells, higher transcript levels in NP over FAT cells for *P53*, *P73*, *Ki67*, *Glis1*, *Cav1*, and *Lif1R* reflect this on a molecular level. Caveolin-1 (Cav1) can stimulate Akt signaling and increase glycolysis in cancer cells. It was suggested that low *Cav1* expression correlates with high reliance on glucose metabolism (Nwosu et al., 2016). Hence, our 2D transcription data suggest that NP cells depend less on glucose than FAT cells and, therefore, would be at an advantage in the NP niche. Similarly, the expression of the cell proliferation marker *Ki67* in standard and degenerate conditions alongside a lower percentage of senescent cells, as demonstrated by SA-β galactosidase staining in the NP lineage in 3D, also suggests a higher proliferation rate for NP cells.

Considered the two most abundant circulating carbon metabolites, glucose (5 mM) and lactate (1 mM) can be interconverted *via* glycolysis and gluconeogenesis (Rabinowitz and Enerback, 2020). The lower senescence rate of NP cells (7.15%) compared to FAT and AF cells (both ∼21%, AF data not shown) at 40 mM lactate further supports that NP cells are well adapted to the NP niche environment. AF cells challenged with 20 mM lactate showed modest decrease in viability in a similar experiment (Wang et al., 2021a). It was shown that lactate acts as a carbon source for various cell types (Hui et al., 2017; Wang et al., 2021a). Based on our existing data, we propose that NP cells in their unique niche developed an efficient metabolic adaption catering to the use of lactic acid over glucose and the Krebs cycle. High glucose might interfere with shared enzymes involved in these metabolic reactions. To further elevate lactate from waste to fuel status, it was recently shown that rabbit AF cells metabolize NP-produced lactate (Wang et al., 2021a). Recent findings in rat NP cells demonstrated that lactate-derived pyruvate is also introduced into the Krebs cycle (Silagi et al., 2020). In this context and given the increasing recognition of lactate as a metabolic driver (Warburg et al., 1927; Rabinowitz and Enerback, 2020; Wang et al., 2021a; Chen et al., 2021), maybe a reciprocal *in vivo* scenario might be envisioned where inner AF cells could fuel NP cells through glycolysis and lactate production.

### 4.2 Stromal cells *in vitro* uphold lineage features

Cells in the IVD and FAT are of mesoderm origin, yet terminal differentiation follows different paths. Advanced omics technologies in IVD research allow for transcriptome profiling at a single and cell population average level (Hickman et al., 2022). Despite the lack of natural signaling cues in monolayer culture, transcripts of the three cell lines (NP, AF, and FAT) in early 2D culture reflect their mesoderm origin alongside their lineage profile through overlapping and distinct molecular signatures. Our *in vitro* transcriptome data largely support biomarkers previously identified for NP and AF cells *in vivo* (Li et al., 2019a; Li et al., 2019b), except that *Snap25* was higher in NP over AF cells *in vitro*, which could be a 2D culture effect (Minogue et al., 2010; Li et al., 2019b).

Transcription factors (TF) are primary regulators of gene expression. Their profile indicates cell lineage commitment and responses to environmental cues. As protagonists of chondrogenesis, the Sox-trio (Sox5, Sox6, and Sox9), hedgehog, and Tgfβ signaling antagonize adipogenesis (Lefebvre et al., 2001; Ikeda et al., 2004; Lee et al., 2017; Zhou et al., 2019). Transcripts of the Sox-trio along those of Shh-mediators *Gli1* and *Gli2* were higher in NP cells along with *Tbx1* transcripts encoding the T-box containing protein brachyury, a clinical marker for chordoma (Dai et al., 1999; Vujovic et al., 2006; Chen et al., 2020), indicating NP cell linage commitment alongside higher transcript levels for *Bapx1* (*Nkx3.2*) an important player in skeletal development (Tribioli and Lufkin, 1999; Lefebvre et al., 2001; Chatterjee et al., 2014; Lee et al., 2017); *Lmx1b* known for its role in dorsal limb mesenchyme patterning (Loomis et al., 1998; Kraus et al., 2001) and *Ntn1* encoding a protein involved in tumor progression and apoptosis control (Mazelin et al., 2004). *Dlx1*/*Dlx2* and *Msx1*/*Msx2* transcripts, regulators of cell lineage commitment during nervous system and bone formation (Kraus and Lufkin, 1999), were lower in NP cells, as was *Pparg*, which is crucial for adipogenesis (Rosen and MacDougald, 2006) and higher in FAT over IVD cells. Surprisingly, in 2D culture, *Runx2* transcript levels were higher in FAT over IVD-derived cells. Runx2 is important for the proliferation of osteoblast progenitors (Kawane et al., 2018), and its loss-of-function in primary chondrocytes facilitates differentiation along the adipogenic lineage *in vitro* (Enomoto et al., 2004). Elevated *Runx2* expression could indicate a phenotypical adaption of FAT cells to the monolayer culture condition as a stretched morphology appears to favor differentiation toward an osteoblast cell type (Lu et al., 2007). *Sox10* transcripts encoding a TF implicated with neural crest and peripheral NS system development acting synergistic with Pax3 were higher in NP cells. However, *Pax3* and *Pax6* transcripts were generally elevated in AF cells along with *Foxn1* encoding a TF that, together with *Pax1*, plays a role in thymus development. Pax1 and Pax9 are well known for their role in IVD patterning (Wilm et al., 1998; Peters et al., 1999; Sivakamasundari and Lufkin, 2012; Sivakamasundari et al., 2013; Sivakamasundari et al., 2017), and *Pax9* was generally downregulated in FAT cells.

Signaling factors are both at the giving and receiving end of differential gene expression. Responding to extracellular cues and channeling the information to the nucleus, they trigger transcriptome changes to direct cell function. Transcripts encoding the semaphorins Sema3b and Sema4d, cell signaling peptides involved in tissue homeostasis (Janssen et al., 2010); Mia, a growth factor involved with ECM organization and neural crest differentiation (Blesch et al., 1994); Gas6 a stimulator of cell proliferation (Gomes et al., 2019); and Tnfsr11b known to inhibit the formation of osteoclasts (Yamaguchi et al., 1998) were all higher in NP over FAT cells. In contrast, transcripts encoding the nerve growth factor VGF; transcripts for Fgf18, a pleiotropic growth factor involved in neurite outgrowth *in vitro*; and *Cxcl3*, a cytokine promoting angiogenesis and connective tissue reorganization, were lower in NP cells, reflecting their non-vascularized and non-innervated source tissue.

Importantly, the mature cells have not lost the ability to produce ECM *in vitro*. Many of the transcripts higher in NP/IVD over FAT cells were associated with the GO terms ECM components, ECM homeostasis, and ECM remodeling, alongside suppression of neurogenesis and angiogenesis, as one would expect given the natural environment of NP cells. *Col1a1*, predominantly in the AF *in vivo*, was reduced in NP over AF cells and *Col2a1*, predominantly the NP *in vivo*, was higher in NP cells as expected (Li et al., 2019b). Transcripts for proteoglycans (PG) such as aggrecan (Acan) and other large and small proteoglycans remained higher in the NP/IVD over FAT cells in monolayer culture. PGs are heavily glycosylated proteins and crucial ECM components alongside collagens, especially in the IVD (Eyre and Muir, 1976; Bushell et al., 1977)*.* Many ECM-PGs have signaling and biomechanical properties (Wilda et al., 2000; Chen et al., 2017). Being of negative charge and involved in maintaining osmotic pressure, especially the bottlebrush PG Acan, plays an important role in the IVD niche (Bibby et al., 2001; Singh et al., 2009). SLRPs are also bioactive components of the IVD. Ogn, with one of the highest transcript levels in NP over FAT cells, impacts proliferation and apoptosis in fibroblasts (Deckx et al., 2016). Other transcripts with higher levels in NP over FAT but less studied in the context of the IVD ECM were *Anos1*, encoding anosmin-1 associated with tumor progression (Choy et al., 2014), and *Bmper*, encoding for a protein that inhibits Bmp signaling, therefore impacting many important signaling cascades critical in chondrogenesis (Yoon and Lyons, 2004; Manzari-Tavakoli et al., 2022). *Vwa5a* transcripts encoding a tumor suppressor (Zhou et al., 2009) were upregulated in FAT over NP cells. Aside from impacting ECM organization and collagen fibrillogenesis, some of these proteins engage in inflammatory response pathways and the phosphatidylinositol-3-kinase (PI3K)/Akt and mammalian target of rapamycin (mTOR) signaling pathway critical for cell cycle regulation (Ryu and Lee, 2017). Surprisingly, transcripts lower in NP over FAT/AF cells were associated with cell migration, despite NP cells being very mobile in monolayer culture (Kraus et al., 2019b).

Fernandes et al. (2020) were among the pioneers applying single-cell RNASeq to analyze the human IVD, identifying SFRP1, BIRC5*, CYTL1, ESM1, and CCNB2* as AF specific and COL2A1*, DSC3, COL9A3*, COL11A1*, and ANGPTL7 as NP-specific after enzymatic cell isolation and 7–8 day culture in DMEM/F12/10% FBS supplemented with 20 μG/ml ascorbic acid. Our data are derived after non-enzymatic cell isolation and *in vitro* expansion without ascorbic acid. Markers identified by Fernades and marked with an asterisk were also expressed with significant differences between AF/NP cells in our bovine data set, alongside 9/20 AF and 6/20 NP markers of their top 20 lists. Very recent single-cell RNASeq data from human NP cells isolated from healthy and degenerate human NP tissue after a multi-step isolation protocol to clarify chondrocyte fate and differentiation advocates that NC lineage cells evolve and take on the cell fate of different chondrocyte subgroups once the IVD undergoes degeneration (Han et al., 2022). Due to a fundamentally different approach, we cannot directly compare expression patterns. However, our transcriptome data suggest that NP cells, often referred to as chondrocyte-like, indeed share chondrocyte attributes but continue to express the NC cell marker *Tbx1* in 2D and 3D culture, reflecting their NC origin (Herrmann et al., 1990; Tang et al., 2012; Nibu et al., 2013). It remains to be investigated if our procedure of isolating NP stromal cells selects for NCNP cells. A comparison with markers of another recent single-cell RNASeq project highlighting the heterogeneous nature of NP cells is hampered due to their differential expression analysis between NP and inner AF tissue, also referred to as the transition zone (Cherif et al., 2022). In summary, the expression of key transcription and signaling factors, along with ECM components, allowed distinguishing the three lineages based on distinct transcript signatures.

### 4.3 Inflammation and matrix remodeling benefits depend on the environment

Metalloproteinases, such as Mmp, Adam, and Adamst, are important in ECM remodeling and associated with cancer and other diseases (Edwards et al., 2008; Jablonska-Trypuc et al., 2016). Differentially regulated transcripts upregulated in IVD/NP over FAT cells included *Adamdec1*, encoding a secreted catalytic protein involved in cell competition (Yako et al., 2018); *Adam23*, encoding a non-catalytic metalloprotease-like protein involved in cell–matrix and cell–cell interaction; *Mmp3*, encoding an enzyme degrading several collagens, PGs, fibronectin, and laminin (Wan et al., 2021); and *Mmp13*, encoding Mmp cleaving predominant type II collagens. More transcripts were differentially upregulated in FAT cells, such as *Adamst14*, encoding Adamst involved in type I procollagen cleavage (Bekhouche and Colige, 2015); *Mmp9*, encoding an enzyme that degrades type IV and type V collagens and can activate Il1b (Forsyth et al., 1999; Van den Steen et al., 2002); *Mmp12*, encoding an inactivator of Ifnγ (Collison, 2018); and *Mmp23b*, encoding a protein associated with GPCR pathways (Supplementary Table S5). This suggests that *in vitro,* during early 2D culture, the association between generated ECM components and the associated remodeling enzyme is preserved on a transcription level.

ECM remodeling and homeostasis are important aspects of developing and mature organisms. Metalloproteases were expressed by all three cell lines and enabled this process, some specific to the type of ECM molecule they produce. Interestingly, *Adamdec1* upregulated in NP cells is considered to provide a competitive edge when adjacent cells fight for survival and space and could be a further indicator that NP cells did develop unique survival mechanisms (Yako et al., 2018).

Degenerative diseases, such as IVDD, rheumatoid, or osteoarthritis, are of complex origin, including multiple comorbidities and other factors, eventually resulting in a chronic inflammatory pain-inducing stage (Klodzinski and Wislowska, 2018; Swain et al., 2020; Lufkin et al., 2021; Parenteau et al., 2021). Regenerative cell therapies targeting these diseases should not further aggravate such a situation. The generation of pro-inflammatory cytokines, such as Il1β, Il6, and Tnfα, that are linked to pathological pain should be avoided (Zhang and An, 2007). However, cytokines with immune suppressive effects could be beneficial. MSCs are attributed to anti-inflammatory effects (Ghannam et al., 2010), whereas no such evidence was previously available for IVD/NP cells (Lyu, 2022). We provide data based on differential RNA expression analysis in 2D and 3D culture for cells from the same donor that IVD cells, with a focus on NP cells, also have beneficial features.

Cytokines mediate inflammatory responses in various ways. Transcripts for the pro-inflammatory cytokine Il12A, a mediator of graft *versus* host disease (Bastian et al., 2019), Il18 and its receptor, Il21r, and Il1rl1 were lower in NP over FAT cells. Il21 signaling involves the Janus kinase and Signal Transducer and Activator of Transcription (JAK-STAT), phosphoinositide 3-kinase (PI3K), and mitogen-activated protein kinase (MAPK) pathway and is considered to have immune suppressive effects (Leonard and Wan, 2016). The Toll-like receptor Il1rl1 appears to convey anti-inflammatory effects in collagen-induced arthritis (Xu et al., 2008). Moreover, transcripts for the angiogenesis promoting and MSC recruiting stromal cell-derived factor 1 (Cxcl12) were lower in NP cells (Takano et al., 2014), whereas transcripts encoding the cytokine Il6 with impact on inflammation, bone homeostasis, and angiogenesis; Cxcl14, acting as an angiogenesis inhibitor (Hromas et al., 1999; Shellenberger et al., 2004); and Cxcr4, encoding the receptor for Cxcl12, were higher in NP cells. This likely renders monolayer cultured adipose stromal cells preferable over NP-derived cells for cell replacement therapies. However, increased expression of *Tnfα* and *IL1b* was noted in 3D culture in FAT *versus* NP stromal cells under degenerate or chondrogenic conditions, which puts adipose stromal cells in a less favorable position for use in NP niche refurbishment.

### 4.4 Proton sources and pH effect on stromal cells

While the physiological pH of most body fluids is in the neutral range (pH 7.4) (Razaq et al., 2003), the IVD niche is slightly acidic (∼pH 7.1) owing to anaerobic lactic acid fermentation and PG-mediated proton retention. This value drops further in the degenerate stage (∼pH 6.5) (Diamant et al., 1968; Nachemson, 1969; Urban et al., 1977; Bartels et al., 1998; Razaq et al., 2000; Razaq et al., 2003; Urban and Roberts, 2003; Bibby and Urban, 2004; Urban et al., 2004; Bibby et al., 2005; Wuertz et al., 2008; Wuertz et al., 2009; Gilbert et al., 2016; Wang et al., 2021a). The presence of stem or progenitor cells in the IVD was demonstrated previously (Risbud et al., 2007; Kraus et al., 2017), and the acidic microenvironment was generally found to negatively impact cell survival, proliferation, and ECM production. Most studies were conducted for the time frame of about 1 week with HCl/NaOH-mediated pH adjustment analyzing various MSC sources, including subcutaneous FAT, bone marrow, and the IVD from diverse host organisms with effects likely mediated through acid-sensing ion channels (ASICs) (Razaq et al., 2003; Wuertz et al., 2008; Wuertz et al., 2009; Li et al., 2012; Han et al., 2014; Gilbert et al., 2016; Liu et al., 2017). As reviewed recently in detail by Lyu (2022), differences in coping with pH-related and other challenges exist between MSC and IVD cells. However, acidity impairs the function of both cell types. None of these findings are disputed here. However, we did not identify differentially expressed transcripts for ASICs between the three stromal cell lines in standard 2D culture. We noted higher levels of NP transcripts associated with the molecular function of Ca^2+^ binding, supporting a rising interest in ion channel signaling in the IVD (Sadowska et al., 2019).

Lyu further mentioned that insufficient clearance in the IVD results in lactic acid waste accumulation. However, due to sparse information about the response of IVD progenitors, conclusions had to be drawn from MSC (Lyu, 2022). Wang et al. (2021a) suggested that lactic acid clearance, in part, might happen through lactate uptake and metabolism by neighboring AF cells. Our analysis in 3D alginate beads used lactic acid for pH adjustment. With lactic acid as a proton and carbon source, we noted an increase in *Col2a1* transcripts in NP and a decrease in FAT cells. We further noted changes in the expression of key metabolic enzymes as described below and proposed that such an adaption might support the production of OAA in NP cells, a metabolite and signaling molecule linked to longevity (Lehmann et al., 2003; Williams et al., 2009; Martins et al., 2016).

### 4.5 NP cell sustainability through lactic acid recycling with OAA as a longevity factor

Intrigued by the low glucose preference, a natural solute gradient in the IVD (Holm et al., 1981), and the hardiness of NP-derived cells, molecular cues might identify metabolic adaptions that facilitate NP cell survival in the NP niche (Figure 8). The nutrient import starts with members of the solute carrier (Slc) superfamily of transporters. The *Slc2* gene family encodes for Glut integral membrane proteins that facilitate hexose and sugar alcohol transport across membranes (Mueckler and Thorens, 2013). Glut1, encoded by *Slc2a1*, transports glucose, hexoses, glucosamine, and reduced ascorbic acid and is present in NP cells (Rajpurohit et al., 2002; Carruthers et al., 2009). One might assume that levels are tightly regulated as Glut1 haploinsufficiency is pathological in humans, whereas overexpression was linked to tumorigenesis (Yamamoto et al., 1990). Despite the glucose transporter Glut1 being highly expressed in NP tissue (Richardson et al., 2008), recent work has suggested that Glut1 is dispensable and that neither glutamine nor fatty acid oxidation is used as an alternative under low glucose conditions. This further supports the idea of lactate as a key metabolite in NP cells (Hui et al., 2017). While S*lc2a1* transcripts were higher in NP over AF cells, *Slc2a3* transcripts encoding for Glut3, with a high affinity for D-glucose (Manolescu et al., 2007), were higher in FAT cells, indicating and supporting higher dependence of FAT cells on glucose. Important cell conditions affecting PPP activity are a high proliferation rate and the need for NADPH. Numerous studies have revealed a significant upregulation of glucose-6-phosphate dehydrogenase (G6pd) in tumor cells (Kowalik et al., 2017). *G6pd* transcripts were higher in NP over AF cells. The enzyme drives the first rate-limiting step of the oPPP, providing the electron donor NADPH and initiating the conversion of glucose-6-phosphate (G6P) to ribose-5-phosphate (R5P), an important building block for nucleotides and as such nucleic acids, especially in proliferating cells. The noPPP leads to metabolites, including glyceraldehyde-3-phosphate (G3P), that can feed into glycolysis. Transketolase (Tkt) and transaldolase (Taldo) are key enzymes of the noPPP (Jin and Zhou, 2019). *Tktl1* showed the highest transcript levels in NP over AF cells and was higher over FAT cells along with transcripts encoding two fructose-1,6-bisphosphate (F1,6 PP) aldolase isoforms AldoA and AldoB, enzymes that mediate the conversion of F1,6PP to G3P and dihydroxyacetone-phosphate (DHAP) during glycolysis and fructose metabolism (Gamblin et al., 1991). *Eno2* transcripts, encoding for an enolase isoform involved in generating PEP, were lower in NP cells, whereas *Pyruvate kinase* (*Pkm*) transcripts were not affected (Figure 8).

Lactate, a less oxidized form of pyruvate, is classically considered a waste product of glucose metabolism but recently gained attention as a circulating carbon source and metabolic driver in a niche environment (Warburg et al., 1927; Valvona et al., 2016; Hui et al., 2017; Rabinowitz and Enerback, 2020; Wang et al., 2021a; Chen et al., 2021). Transcripts for different isoforms of lactate dehydrogenase (Ldh) were differentially expressed in the three cell types, with NP cells showing higher *LdhD* transcripts. LdhA has a higher affinity to pyruvate over lactate, whereas LdhD converts lactate to pyruvate (LeVan and Goldberg, 1991; Valvona et al., 2016). The Ldh activity is associated with the monocarboxylic transporter protein (Mct) activity, which transports lactic acid and pyruvate across plasma membranes. The transporters are encoded by the *Slc16* gene family (Jitrapakdee et al., 2006). Several Mct encoding transcripts were higher in NP cells, especially *Slc16a2*. Gluconeogenesis-associated fructo-bi-phosphatase 2 (Fbp2) transcripts were over 700-fold lower in NP versus FAT cells, suggesting gluconeogenesis from lactate is not a prominent feature of NP cells. Interestingly, higher transcripts encoding pyruvate carboxylase (Pc) in NP over FAT cells could aid NP cells in refueling the Krebs cycle; however, transcripts for many enzymes converting the various metabolites of the Krebs cycle were lower in NP cells. Similarly, transcripts encoding enzymes that would allow for alternative pathways or shuttle mechanisms were lower in NP cells (Figure 8).

Generated by condensation of pyruvate or PEP with CO_2_, the oxidation of malate or deamination of aspartate, the metabolite OAA wears multiple hats. However, malate conversion to OAA was described as a thermodynamically unfavorable step in the Krebs cycle (Wang et al., 2021a). Being an intermediate in the Krebs cycle and participating in gluconeogenesis, OAA could serve as an energy source. Nevertheless, based on carbon tracing, this does not appear to be its major function in NP cells (Silagi et al., 2020). Our transcriptome-based data, however, suggest that metabolic adaptions in NP over FAT and AF cells might channel some lactate into OAA production. Transcripts for the mitochondrial phosphoenolpyruvate carboxykinase 2 (Pck2) facilitating the conversion of OAA to PEP when producing glucose from lactate were lower in NP over AF and/or FAT cells. *Me3* transcripts encoding a pyruvic-malic carboxylase (malic enzyme) were also lower in NP cells along with *Slc1a3* transcripts encoding the glutamate aspartate transporter 1 (Glast1), whereas transcript levels for ATP-citrate lyase (Acly) and malate dehydrogenase (Mdh) showed no significant difference between the cell lines, altogether supporting reduced importance of mitochondrial activity in NP cells (Gan et al., 2003; Madhu et al., 2020) and an adaption that favors a lactate based metabolism. Since most amino acids were provided through the medium, the need for shuttle mechanisms might not have arisen. Transcripts for glutamate dehydrogenase (Glutd) were not increased in NP cells, nor were those for oxoglutarate dehydrogenase (Ogdh). However, existing levels might be sufficient, or many of those enzymes could be regulated at the protein level. From a transcriptome point, metabolic adaptions in the NP cells appear to channel lactate into OAA production based on higher *Pc* transcript levels in NP cells, encoding pyruvate carboxylase, an enzyme that converts pyruvate to OAA (Figure 8).

An OAA-induced lifespan extension through the FoxO/Ampk signaling pathway was suggested in *C. elegans*. FoxO proteins have an important role in development, are connected to many signaling pathways, and have a complex role in cell longevity and tumor suppression (Lehmann et al., 2003; Williams et al., 2009; Martins et al., 2016). OAA can trigger *Fox* expression, which impacts P53/P21 signaling and possibly enables NP cells to survive under NP niche conditions (Figure 9). Fox-TFs showed higher transcript levels in NP cells, including *FoxO4* alongside *Prkaa2*, encoding a subunit of the AMP-activated protein kinase (Ampk) in vertebrates (Hardie and Carling, 1997). *Fam183A*, the top downregulated transcript in NP over AF cells, is speculated to be a target for FoxO4 binding. *FoxP2* is associated with Wnt, Hedgehog, and Notch signaling. FoxF2 has inhibitory effects in many tumors and might bind to promoters of ECM-related genes such as *Acan* and *Col2a1* during vertebrate development (He et al., 2020), both NP biomarkers *in vitro* and *in vivo* (Li et al., 2019b). FoxC2 is a TF involved in mesenchyme development and tumor progression (Mani et al., 2007). FoxA3 is associated with glucose metabolism homeostasis and longevity (Ma et al., 2014). *FoxL*1 was the most upregulated *Fox* transcript in IVD over FAT cells. Although *FoxL1* overexpression is associated with gliomas (Chen et al., 2019), the FoxL group of TFs is less well studied, but a connection between FoxL1 and cell survival was established through the PI3K/Akt/mTor signaling pathway, which was impacted upon through the differential expression of ECM molecules such as Col9a1 (high in NP cells) and Col1a1 and LamC2 (both lower in NP cells) (Ryu and Lee, 2017). Crosstalk between Fox-TFs and P53 was suggested (Dijkers et al., 2000; You and Mak, 2005). In this context, we found that *P53* and *P73* transcripts at least doubled in IVD over FAT cells. As a tumor suppressor, P53 protects cells by controlling progression through the cell cycle and allowing DNA repair (Figure 9) (Levine, 1997; May and May, 1999). Cell cycle arrest is arranged through transcriptional activation of P21 by arresting cells in G1, promoting differentiation over proliferation (Harada and Ogden, 2000). *P21* was not differentially expressed in 2D. In adult stem cells, reduced P53 aids in reprogramming (Marion et al., 2009; Maeso-Alonso et al., 2021), whereas P73 aids in controlling cell cycle progression and plays additional rolls in cell–adhesion and cell–ECM interactions (Maeso-Alonso et al., 2021). A positive correlation between *P53*, *P73*, and *Ki67* transcript levels was noted in all cell types, with higher levels in IVD over FAT cells, whereas P53-induced protein 11 transcripts (*TP53I11*, *Pig11*) (Gu et al., 2021) showed a negative correlation and were downregulated in IVD over FAT cells. *Perp* encoding an effector in the TP53-dependent apoptotic pathway was also downregulated in NP over FAT cells in 2D culture. In this context, the upregulation of *Ki67*, a cell proliferation marker with gradually increasing transcript and protein levels throughout the cell cycle (Li et al., 2021), in NP over FAT cells might indicate that *P53/P73* expression in IVD cells is not inducing the apoptotic pathway; instead, it is slowing down cell cycle progression, potentially facilitating somatic cell reprograming (Figure 9). *Ros1* transcripts for a proto-oncogene upregulated in many tumor cell lines were lower in NP over FAT cells, suggesting that increased proliferation in NP cells is not due to an aberrant phenotype.

Ideally, OAA concentration measurements would support our findings. However, currently available enzymatic tests are not suitable for using DMEM-based medium according to the manufacturer’s instructions (BioAssay Systems). In this context, a detailed metabolic assessment studying lactate efflux through carbon isotope labeling in rat NP cells indicated, based on isotope recovery in glutamate, that the majority of lactate-derived pyruvate is introduced into the Krebs cycle via Pdh and acetyl-CoA without detectable Pc and OAA contribution to glutamate production (Silagi et al., 2020). This challenges our transcriptome-based findings, which identified higher *Pc* transcripts in NP over FAT cells *in vitro*; yet reduced transcription of enzymes of the PDH complex and Krebs cycle or Pck. In support of our hypothesis that OAA could promote longevity in NP cells, the differential expression of transcripts for the aforementioned metabolic enzymes, along with differential *Fox* gene expression and a generally lower proportion of senescent cells among NP-derived stromal cells in all tested conditions, suggests such a mechanism as feasible. Therefore, we suggest that molecular mechanisms for the generation of OAA as a signaling molecule through extended lactate metabolism could be in place in NP cells. This could be a minor contribution irrelevant or not favored for glutamate synthesis. OAA was recognized as a bioenergetic medicine agent to treat neurodegenerative diseases (Wilkins et al., 2014). OAA, as a signaling molecule, could provide survival benefits to NP progenitor cells that adipose stromal cells lack. There was no indication of the OAA-*Fox* connection in FAT cells from the 2D differential expression analysis and the higher proportion of senescence seen in FAT cells might pose a challenge when using adipose stromal cells to repopulate a degenerate NP niche. This finding is supported by recent studies in rats (Han et al., 2014) and the abovementioned single-cell RNASeq data from healthy and degenerated human NP cells recommending the use of NP progenitor cells for IVDD treatment (Han et al., 2022).

### 4.4 Stemness and somatic reprogramming

Stem cell presence has been suggested for most vascularized tissue of the body (Crisan et al., 2008), yet the NP is avascular. We have previously demonstrated that NP stromal cells have chondrogenic and osteogenic potential (Kraus et al., 2017). In the larger context of cell reprogramming, CD markers can provide information about the differentiation status of a cell line. CD markers are based on cell surface proteins initially used for immunophenotyping (Bernard and Boumsell, 1984). Over time, many were characterized in their function and helped characterize mesenchymal stem or stromal cells (Dominici et al., 2006; Lv et al., 2014). Minimum criteria of MSCs include plastic-adherence under standard culture conditions alongside the expression of CD105, CD73, and CD90 and the absence of CD45, CD34, CD14 or CD11b, CD79a or CD19, and HLA-DR and the ability to differentiate into the three-mesoderm lineage *in vitro*. Other surface antigens generally expressed by MSCs include CD10, CD13, CD29, and CD44 (Dominici et al., 2006; Lv et al., 2014). The NP, AF, and FAT stromal cells investigated here were isolated based on their ability to attach to an untreated plastic surface. NP cells showed higher transcript levels for the CD90 (Thy1) cell surface glycoprotein and lower levels for *CD34* transcripts, whereas other markers were not differentially expressed between the cell lines. Increased *Thy1* expression for NP over AF cells *in vitro* differs from our previous observations *in vivo* (Li et al., 2019b). Higher transcript levels in the NP were also found for *CD101* encoding the transmembrane glycoprotein V7, a prognostic marker for gliomas (Rivas et al., 1995; Liu et al., 2022a), and *CD109* encoding a protein that negatively regulates TGFβ signaling and is involved in tumorigenesis (Li et al., 2016; Lee et al., 2020). Transcript levels higher in FAT cells included those encoding CD36, a glycoprotein receptor involved in cytokine and inflammasome production (Stewart et al., 2010); CD200 with a role in immunosuppression and regulation of anti-tumor activity (Khan et al., 2021); CD248 (endosialin) with predicted involvement in migration and ECM binding (Rettig et al., 1992); and CD300, a hypoxia-inducible regulator of VEGF production involved in immune response (Raggi et al., 2014; Cao et al., 2021). Aldh enzymes oxidize aldehydes to carboxylic acids, yet some members are discussed as cancer stem cell markers (Nakahata et al., 2015). Several transcripts we found higher in FAT over IVD cells. A benefit of adult stromal cells is their multipotent over pluripotent differentiation potential, a restriction rendering them less tumorigenic than induced pluripotent or embryonic stem cells, however, potentially hampering their plasticity in replacing ailing cells (Pittenger et al., 1999; Kraus et al., 2021). *Oct4* is known as a stemness and reprogramming marker but also has a role in lineage specification (Zeineddine et al., 2014). We did not see transcripts for the pluripotency factor *Oct4* differentially expressed between the cell types in 2D culture. However, transcripts for its partner Sox2 were higher in NP and FAT over AF cells. *Sox2* encoded one of the reprogramming factors important for stem cell maintenance and cell fate determination (Takahashi et al., 2007; Feng et al., 2009) and was suggested as a biomarker to distinguish between NP and AF cells *in vivo* (Li et al., 2019b). We also noted an increase in *Lif* expression in NP and FAT over AF cells and increased transcripts for the Lif receptor in NP over FAT cells. Lif signaling is considered crucial for maintaining pluripotency in naïve stem cells. Interestingly, Sox2/Oct4 downstream, such as *Klf4* and *Top2a*, were slightly higher in NP over FAT cells, whereas *Mras* was slightly higher in FAT over IVD cells (data not shown) (Rizzino, 2013; Zeineddine et al., 2014).

*Glis1*, a zinc finger TF, recognized as a somatic cell reprogramming factor involved in cell differentiation by restricting lineage commitment of multipotent cells (Maekawa et al., 2011; Scoville et al., 2017; Jetten, 2018; Li et al., 2020) could be a key player in NP cell phenotype adaptions. Glis1 showed the highest transcript levels in NP over FAT cells, and transcripts higher in NP over FAT cells were enriched for DNA binding transcription activator activity. This suggests a more progressive role of NP cells in adapting to the 2D environment in a standard medium through active reprogramming. High levels of *Glis1* in NP over FAT cells and the presence of *Glis1* in a subpopulation of NP cells *in vivo* in combination with increased transcripts for other reprogramming affiliated markers further suggest a higher progenitor cell potential in NP over FAT cells. Expression of *Glis1* remained higher in NP over FAT cells in 3D culture in standard medium, but *Oct4* levels were higher in FAT cells, perhaps due to a structurally influenced component triggering somatic cell reprogramming of adipose cells (Figure 4E, Figures 10A,C). *P53* levels remained higher, and *P21* levels were lower in NP cells in 3D culture. P53 is primarily considered a transcriptional activator, with transcriptional repression mediated through its target P21, among other mechanisms (Sullivan et al., 2018). Increased P21 levels could be one reason for a higher proportion of senescent cells in the FAT cell lineage in 3D culture as identified by SA β-galactosidase staining (Figures 10A,D). Transcript levels for the inflammatory markers Tnfα and Il1b were not significantly different between the two cell lines in 3D culture under standard conditions (Figure 10A).

### 4.5 Lactylation and other somatic reprogramming in 3D stromal cell culture

Monolayer culture and adherence to plastic do not reflect the typical environment of mammalian cells, which are usually embedded in 3D ECM of various stiffness, composition, and density. Bio-scaffold development, including the use of alginate, has proven helpful in the field of IVD research (May et al., 2020). Cell shape and surface stiffness are important determinants for adipose stem cell differentiation into a chondrogenic phenotype. Stem cells maintained with a round morphology differentiated into adipocytes even in osteogenic media (Lu et al., 2007). Morphological changes from flat to round in the 3D alginate-based environment could be seen by histological staining, and a BISH protocol for the analysis of selected transcripts was developed. Upon 3D culture using standard D10^S^L media, *Col2a1* and *Krt19* expression along with TFs such as *Tbox* and *Noto* were now higher in FAT over NP cells indicating adjustment of adipose stromal cells to matrix cues of a scaffold that was developed for IVD cell culture (Maldonado and Oegema, 1992; May et al., 2020). *Noto*, a homeobox TF acting downstream of FoxA2 and brachyury (*Tbox*) during the NC development can direct induced pluripotent stem cell-derived progenitor cells toward an NC fate (Colombier et al., 2020). *Noto* showed no significant difference in 2D, suggesting some form of somatic cell reprogramming upon transitioning from 2D to 3D.

FAT is a convenient source for autologous stem/stromal cells in regenerative medicine. However, the degenerated NP offers a harsh environment for settlement (Lyu, 2022). Some attributes of the degenerate NP were partially mimicked through lactic acid at a concentration of ∼40 mM. This is higher than plasma levels (1 mM) and higher than previously described for the center of an IVD (16 mM) (Bartels et al., 1998; Sugimoto et al., 2012; Hui et al., 2017; Wang et al., 2021a). The presence of lactic acid could impact cells in several ways: a breakdown of ECM molecules, as described *in vivo*, could modify ECM signaling cues, lactic acid as a carbon source could impact cell metabolism, and lactylation could promote epigenetic cell reprogramming. As a novel concept, somatic cell-reprogramming through lactylation or lactate-induced histone modification recently moved into the spotlight (Zhang et al., 2019). Metabolic reprogramming is best studied in cancer cells as an adaption to hypoxia and hypo-nutrient conditions (Yoshida, 2015; Zhang et al., 2019; Chen et al., 2021), a condition all too familiar for NP cells. NP cells are in possession of molecular tools for lactate transport and metabolism. Adding lactic acid to 3D culture significantly increased *Glis1* expression in FAT cells beyond levels in NP cells, suggesting Glis1 as a mediator of lactylation-based somatic cell reprogramming. We do not claim that the stromal cells undergo reprogramming toward a pluripotent state. Somatic cell reprogramming and cancer development share a metabolic switch toward glycolysis, and concern exists based on data from induced pluripotent cells that partial reprogramming could be tumorigenic (Folmes et al., 2011; Ohnishi et al., 2014). It is unclear if partial somatic reprogramming might negatively impact stromal cell performance. We noticed an increase in tumor-promoting or a decrease in anti-tumorigenic transcripts, alongside an increased inflammatory potential associated with high *Glis1* transcript levels in 2D. Glis1, a P53-independent somatic reprogramming factor, promotes but is not required for Oct4/Sox2/Klf4- (OSK-) dependent reprogramming (Maekawa et al., 2011; Scoville et al., 2017), which could explain why the relative *Oct4* and *Glis1* transcript levels vary depending on the culture condition. Based on *Glis1/Sox2* expression in 2D, we propose that NP stromal cells have a higher degree of stemness than FAT stromal cells, but a 3D environment and exposure to lactic acid appear to facilitate stemness in FAT cells and might elevate the risk of tumorigenesis for FAT stromal cells in the degenerate NP niche as indicated by higher *Tbox* (*Tbx1*) levels, a prognostic chordoma marker. Initiation of somatic reprogramming is further indicated by a now higher expression of *Col2a1* and *Pax1.* NP cells find themselves under “homeroom” conditions, prompting no significant changes in *Glis1* but increased *Oct4* levels. *P53*, *P21*, and *Ki67* transcripts levels agree with the concept of transcriptional activation of *P21*, leading to G1 arrest that would promote differentiation over proliferation, as in FAT cells reduced P21 expression upon lactic acid exposure correlated with increased Ki67 expression, indicating cell proliferation (Harada and Ogden, 2000; Marion et al., 2009; Maeso-Alonso et al., 2021) (Supplementary Figure S1). For glucose/lactate metabolism-related enzymes, a significant increase in *LdhA/B* was noted in FAT over NP cells (Figure 10A, Supplementary Figure S1). Possibly other Ldh isoforms, as seen in the transcriptome profiling of cells in 2D culture, could play a role here. Inflammatory markers, *Tnfα* and *Il1b*, were significantly higher in FAT over NP cells in these limited NP niche conditions (Figure 10A, Supplementary Figure S1).

Although the NP cells are of NC origin, they are often considered chondrocyte-like (Trout et al., 1982b; Hunter et al., 2004). Hence, media for IVD cells is often supplemented with ascorbic acid and proline (Kraus et al., 2017). Ascorbic acid (vitamin C), among several functions, is involved in collagen biosynthesis on a transcriptional and posttranslational level (Pinnell, 1985; Combs and McClung, 2016), and proline is an important constitute of collagen (Karna et al., 2020). Glut1 transporters enable the glucose and ascorbic acid to pass the plasma membrane. Although *Col2a1* transcript levels were similar between NP and FAT cells in chondrogenic (D10^C^L) conditions, they were at their highest for NP cells (Supplementary Figure S1). *Krt19*, *Noto*, *Tbox*, and *Pax1* expression was increased in FAT over NP cells. From a metabolic perspective, transcript levels for *Gys1*, *Mdh*, and *LdhB* were significantly lower in NP over FAT cells. The proportion of senescent cells remained higher in FAT cells. However, for both cell lines, the highest proportion of senescent cells and *P21* transcript levels was noted under chondrogenic conditions (Figure 10A, Supplementary Figure S1). *Glis1* expression was slightly higher in FAT over NP cells. Transcript levels for the inflammatory markers, *Tnfα* and *IL1b*, were significantly higher in FAT over NP cells (Figure 10A). Chondrogenic conditions triggered somatic reprogramming in both cell lines, potentially mediated through the presence of ascorbic acid as recently a role of ascorbic acid in epigenetic reprogramming was suggested (Young et al., 2015; Liu et al., 2022b). Aside from an increase in *P21* transcripts, it is unclear what caused the increase in senescence in both cell lines. A high senescence rate might counteract some potentially deleterious tumorigenic reprogramming effects but would likely impact the efficiency of transplanted cells to contribute to a chondrogenic niche. In summary, we propose that changes in extracellular (lactic) acid promote cell type-dependent Glis1-mediated somatic cell reprogramming in NP and FAT-derived stromal cells.

## 5 Conclusion

Ideally, cells used for cell therapy in IVDD can maintain themselves without disturbing the unique cell/ECM relation, can replenish the correct ECM macromolecules, and will not further aggravate the condition, simply hanging in there and doing the job for as long as possible. It is not an easy task in an unwelcoming environment and not an easy task to assess, given the nature of the IVD. Herein, we aimed to understand the ability of IVD and adipose stromal cells to serve the IVDD niche from a transcriptome point of view. While adipose stromal cells have the benefit of accessibility and can adapt to a NP-cell like phenotype, key differences remain. Lactic or ascorbic acid appears to reprogram stromal cells through *Glis1*. High levels of *Glis1* appear to be associated with an increased risk for inflammation and tumorigenesis. 3D adipose stromal cells show a higher risk of aggravating inflammatory conditions and a higher senescence rate compared to NP cells. We further propose that NP cells developed mechanisms to use lactic acid to their advantage, potentially even for “in-house” production of a longevity supplement.

## Data Availability

The original contributions presented in the study are publicly available. This data can be found in NCBI GEO (https://www.ncbi.nlm.nih.gov/geo/), accession number GSE216377.

## Supplementary material

Supplementary Figure S1 Bar graphs indicate RNA levels after BISH and ImageJ analysis of NP and FAT cells after 10 days of 3D alginate bead culture under different culture conditions for genes related to the nucleus pulposus (NP) (yellow), glucose and lactate metabolism (orange), inflammatory markers (green), cell proliferation and senescence markers, and SA-beta galactosidase staining (blue). Cells were isolated from a single donor to avoid the genotype effect. A minimum of 25 cells were analyzed after subjecting three beads per probe, cell line, and condition to BISH. S, standard media D10^S^L; D, media D10^D^L mimicking degenerate conditions; C, chondrogenic D10^C^L media. Significance is indicated as *** (*p* < 0.001); **(*p* < 0.01); *(*p* < 0.05).

Supplementary Figure S2 Top three Gene Ontology (GO) categories after functional enrichment for molecular or biological functions.
