# Supplementary figures and images for: Glis1 and oxaloacetate in nucleus pulposus stromal cell somatic reprogramming and survival
Source: Front Mol Biosci. 2022 Nov 3;9:1009402. doi: 10.3389/fmolb.2022.1009402 (PMC9671658; doi:10.3389/fmolb.2022.1009402)

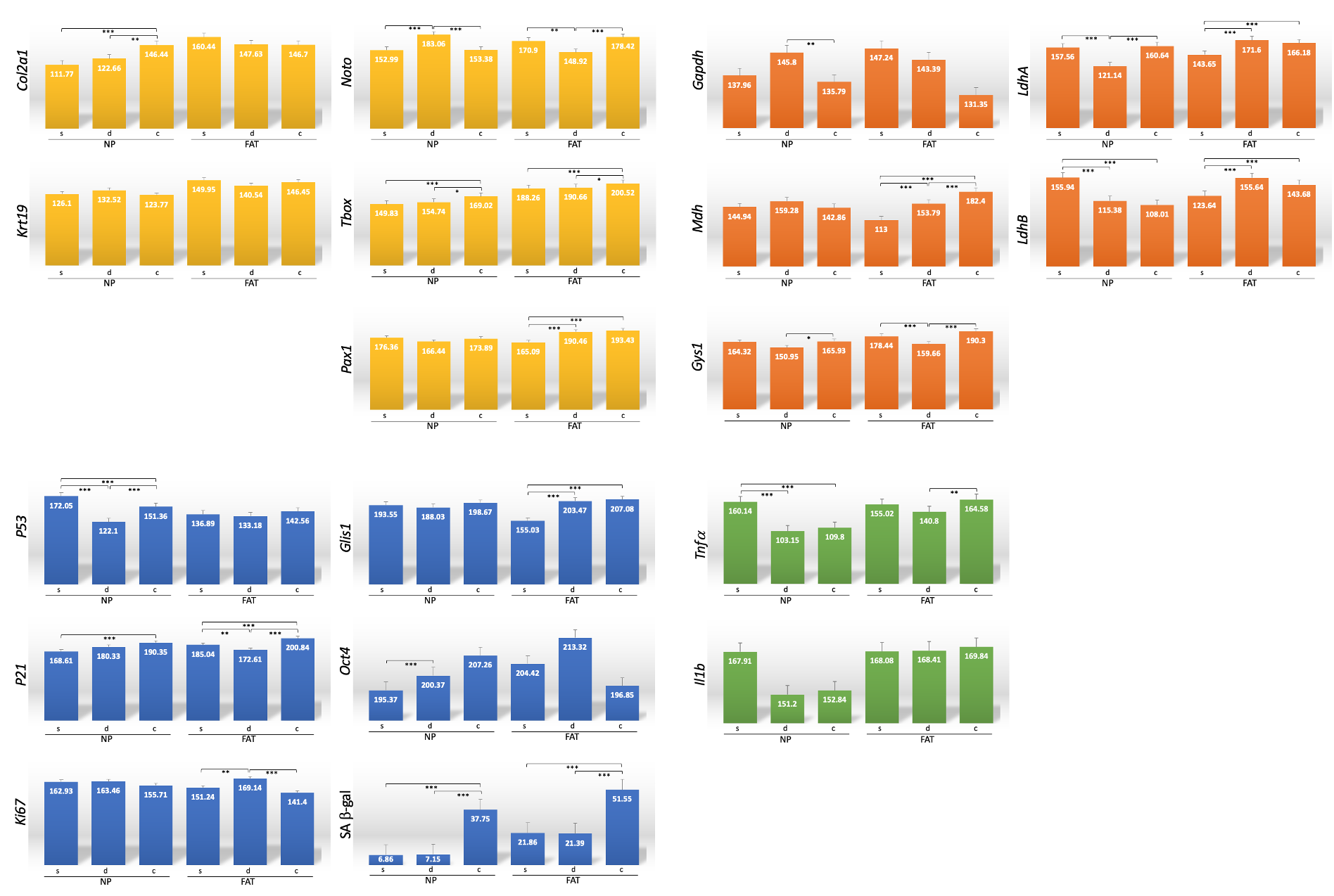

Supplement: Supplementary file 1 [file Image1.TIFF]

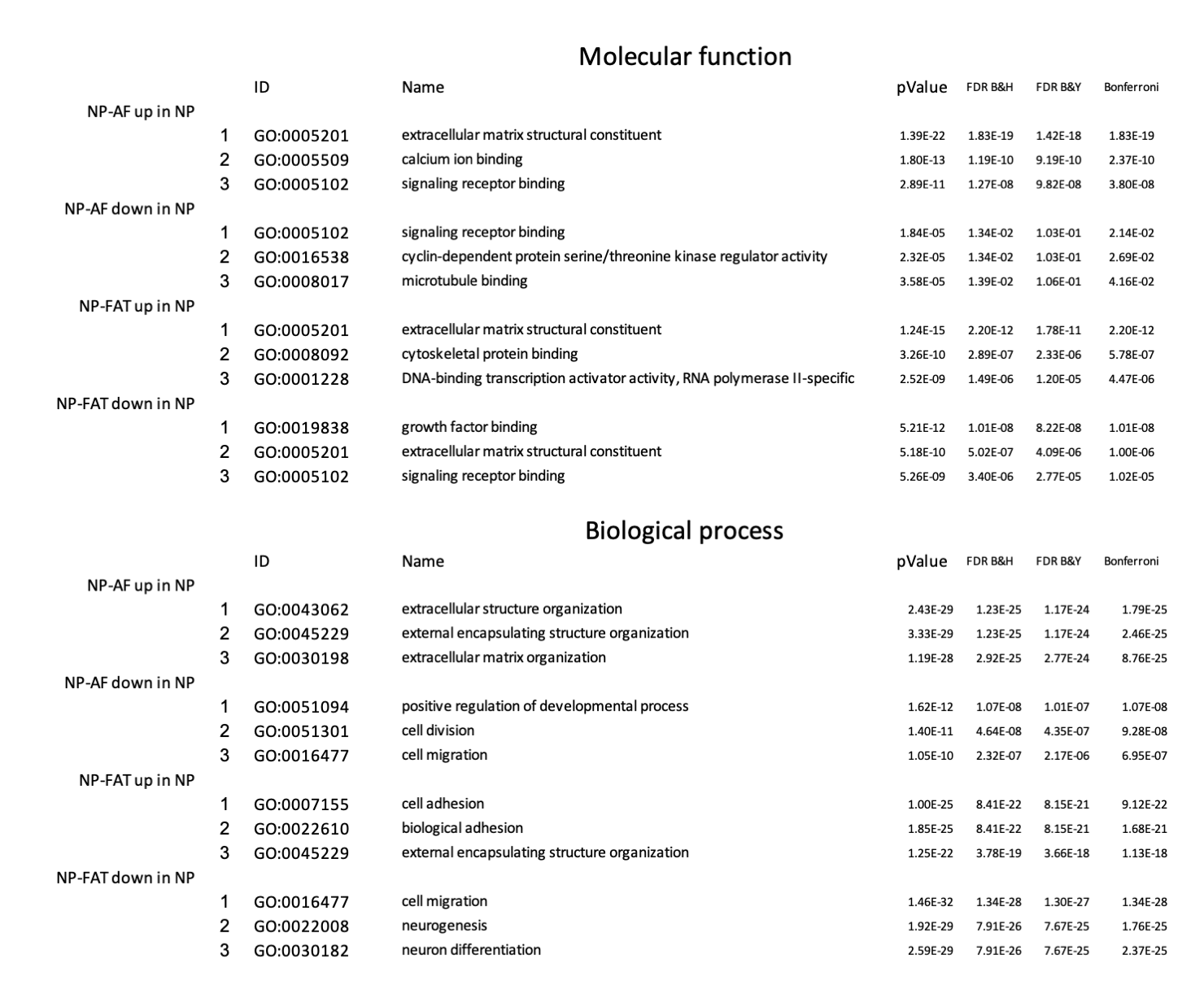

Supplement: Supplementary file 7 [file Image2.TIFF]
