# Supplementary material for: Glis1 and oxaloacetate in nucleus pulposus stromal cell somatic reprogramming and survival
Source: Front Mol Biosci. 2022 Nov 3;9:1009402. doi: 10.3389/fmolb.2022.1009402 (PMC9671658; doi:10.3389/fmolb.2022.1009402)
Supplement: Supplementary file 2 [file Table1.DOCX]

**Supplementary Table 1:** Differentially expressed genes between annulus fibrosus (AF) and nucleus pulposus (NP) cells of the IVD and adipose (FAT) stromal cells of the same donor associated with the functional enrichment term extracellular matrix structural constituent (GO:0005201) displayed by the log2 fold changes (FC[log2]).

| Extracellular matrix structural constituent (GO:0005201) | | | | | | | | | | | |
| --- | --- | --- | --- | --- | --- | --- | --- | --- | --- | --- | --- |
| AF-NP | | | | FAT-NP | | | | FAT-AF | | | |
| high in NP | FC[log2] | low in NP | FC[log2] | high in NP | FC[log2] | low in NP | FC[log2] | high in FAT | FC[log2] | low in FAT | FC[log2] |
| ABI3BP | 1.8189442 | ADIPOQ | 1.91455067 | ABI3BP | 8.110245785 | AGRN | 1.297649049 | AGRN | 1.56498468 | ABI3BP | 6.298484099 |
| ACAN | 1.667282 | COL1A1 | 3.04885429 | ACAN | 9.828100009 | COL13A1 | 6.709421709 | ASPN | 1.71285826 | ACAN | 8.167513169 |
| ANOS1 | 8.108303 | COL5A3 | 2.62758723 | ADIPOQ | 1.261953294 | COL18A1 | 5.150759135 | COL12A1 | 1.47481445 | ADIPOQ | 3.184899876 |
| ASPN | 2.1953102 | COMP | 2.71207347 | ANOS1 | 7.970139049 | COL1A1 | 3.909171674 | COL13A1 | 4.0802259 | BGN | 1.14160495 |
| BMPER | 3.645375 | EMILIN2 | 2.31062043 | BGN | 1.410500522 | COL1A2 | 1.278068259 | COL18A1 | 5.82756168 | BMPER | 4.685229826 |
| CHADL | 4.717299 | FBLN1 | 2.91051851 | BMPER | 8.351942486 | COL4A1 | 8.844559266 | COL3A1 | 1.79636485 | CHI3L1 | 4.150201644 |
| CHI3L1 | 3.2721062 | LAMA3 | 2.49166258 | CHAD | 2.417836605 | COL4A2 | 8.38440299 | COL4A1 | 11.2388172 | COL11A1 | 1.719380525 |
| COL11A1 | 1.6627698 | LTBP1 | 2.30822643 | CHADL | 3.918997539 | COL5A1 | 1.394863932 | COL4A2 | 10.8906659 | COL11A2 | 2.612360794 |
| COL11A2 | 3.5673083 | LTBP4 | 1.58075042 | CHI3L1 | 7.425008316 | COL5A3 | 6.528369844 | COL5A1 | 1.42416869 | COL14A1 | 1.173413094 |
| COL12A1 | 1.5428026 | MFAP5 | 1.96617338 | COL11A1 | 3.379606517 | COL6A5 | 6.602317126 | COL5A3 | 3.89928925 | COL15A1 | 1.829606129 |
| COL21A1 | 2.7009959 | MFGE8 | 1.8226921 | COL11A2 | 6.194222039 | CTHRC1 | 1.235152929 | COL6A5 | 5.53244341 | COL8A1 | 3.780821286 |
| COL24A1 | 1.4931738 | PODN | 2.10479293 | COL14A1 | 1.595169175 | DPT | 4.590611599 | CTHRC1 | 1.35955814 | COL8A2 | 2.269324643 |
| COL27A1 | 1.9903611 | TFPI2 | 4.77425045 | COL15A1 | 1.165417276 | ECM1 | 1.198751775 | DPT | 4.11825533 | COL9A2 | 2.261675771 |
| COL2A1 | 11.01776 | TINAGL1 | 2.2882352 | COL20A1 | 2.464437603 | EMILIN2 | 8.297428739 | ECM1 | 1.45861223 | COMP | 2.694286388 |
| COL3A1 | 1.4501463 | VCAN | 1.13684359 | COL21A1 | 5.285588908 | FBLN1 | 6.426568175 | EMILIN1 | 1.04470348 | CRELD1 | 1.046190005 |
| COL4A1 | 2.3962921 | VWA5A | 3.75426287 | COL27A1 | 1.806013308 | FBLN2 | 1.788727016 | EMILIN2 | 5.98922539 | EDIL3 | 5.052010031 |
| COL4A2 | 2.5016019 |  |  | COL2A1 | 11.15022874 | FBLN5 | 2.782418004 | FBLN1 | 3.51958586 | ELN | 2.89969249 |
| COL4A5 | 3.0669769 |  |  | COL4A5 | 2.330631316 | FN1 | 1.196875272 | FBLN2 | 2.77357377 | FBN2 | 3.100372245 |
| COL8A2 | 1.3536622 |  |  | COL4A6 | 2.722905919 | IGFBP7 | 1.772685996 | FBLN5 | 1.95111085 | FGL2 | 2.357872758 |
| COL9A1 | 5.0592177 |  |  | COL6A3 | 1.005087714 | LAMA1 | 4.645901346 | FBN1 | 1.57566991 | FMOD | 2.517352765 |
| COL9A2 | 4.6481098 |  |  | COL8A1 | 4.500512675 | LAMA2 | 4.425873185 | FN1 | 2.06032301 | HAPLN1 | 8.346758487 |
| COL9A3 | 2.7212343 |  |  | COL8A2 | 3.614236833 | LAMA3 | 2.722162164 | IGFBP7 | 3.27417967 | LAMA5 | 3.032882934 |
| DCN | 1.1075333 |  |  | COL9A1 | 9.477479704 | LAMA4 | 1.722840085 | LAMA1 | 2.65224289 | LAMB3 | 2.375190175 |
| EDIL3 | 1.0971039 |  |  | COL9A2 | 6.87421308 | LAMC2 | 5.646950621 | LAMA2 | 4.49808106 | LTBP4 | 1.566543026 |
| EFEMP1 | 3.3330155 |  |  | COL9A3 | 3.624990538 | LTBP1 | 2.142539673 | LAMA4 | 3.8842463 | MATN2 | 2.740346625 |
| EMILIN1 | 1.3898188 |  |  | CRELD1 | 1.579794962 | LTBP2 | 1.004304728 | LAMB1 | 1.16449986 | MATN4 | 2.756958209 |
| FBN2 | 2.6519749 |  |  | DCN | 1.718970313 | MFAP4 | 4.094790266 | LAMC2 | 5.5664876 | MGP | 2.8587377 |
| FGL2 | 1.3650751 |  |  | EDIL3 | 6.148525006 | MFAP5 | 8.572471869 | MFAP4 | 3.50164883 | NTN1 | 3.078973096 |
| HAPLN1 | 1.7876548 |  |  | EFEMP1 | 3.998377154 | MFGE8 | 1.847346949 | MFAP5 | 6.59076782 | OGN | 2.083623786 |
| HMCN1 | 1.833632 |  |  | ELN | 1.941756505 | MXRA5 | 5.258470759 | MXRA5 | 4.46387344 | OPTC | 4.255888276 |
| HSPG2 | 1.4795682 |  |  | FBN2 | 5.723284224 | PAPLN | 1.23383463 | POSTN | 3.23136977 | PRELP | 1.782394154 |
| IGFBP7 | 1.5012489 |  |  | FGL2 | 3.718253237 | PODN | 2.211942994 | SLIT2 | 7.39584719 | PRG4 | 3.148890779 |
| LAMA4 | 2.1636911 |  |  | FMOD | 2.498794367 | POSTN | 3.513622594 | SPARC | 1.46755298 | SPON1 | 1.908104158 |
| LAMB3 | 2.0781635 |  |  | HAPLN1 | 10.13134477 | SLIT2 | 3.807130615 | SPOCK1 | 8.01509231 | THBS3 | 1.460414261 |
| LAMC1 | 1.0484206 |  |  | HMCN1 | 2.578196208 | SLIT3 | 5.459798197 | SPOCK2 | 1.42548109 |  |  |
| MATN3 | 1.9298704 |  |  | LAMA5 | 3.443152753 | SPOCK1 | 7.981662918 | SRPX2 | 1.24431358 |  |  |
| MATN4 | 1.0013228 |  |  | LAMB3 | 4.4477457 | SPOCK2 | 1.654609007 | TFPI2 | 2.60303012 |  |  |
| NPNT | 3.6093636 |  |  | LUM | 1.05085216 | TFPI2 | 7.38021211 | TGFA | 2.29648404 |  |  |
| NTN1 | 1.0639745 |  |  | MATN2 | 2.316752528 | TGFBI | 6.586150327 | TGFBI | 7.52074968 |  |  |
| OGN | 3.6067065 |  |  | MATN4 | 3.766956617 | THBS2 | 1.052405941 | TGFA | 2.29648404 |  |  |
| OPTC | 2.1179216 |  |  | MGP | 3.653613596 | TINAGL1 | 8.080938089 | THBS2 | 1.28309835 |  |  |
| PRELP | 0.9892352 |  |  | NPNT | 4.704111588 | TUFT1 | 1.444146435 | TINAGL1 | 5.79109761 |  |  |
| PRG4 | 4.9892352 |  |  | NTN1 | 4.136505763 | VCAN | 2.448510774 | TUFT1 | 1.95458972 |  |  |
| SCARA3 | 1.4794332 |  |  | OGN | 5.689254872 | VWA5A | 7.109704917 | VCAN | 1.30802741 |  |  |
| THBS1 | 2.5175927 |  |  | OPTC | 6.372923173 |  |  | VWA5A | 3.35206562 |  |  |
| TMEFF2 | 1.4471184 |  |  | PRELP | 4.16529868 |  |  |  |  |  |  |
| VWA1 | 1.3803524 |  |  | PRG4 | 8.136752539 |  |  |  |  |  |  |
|  |  |  |  | SCARA3 | 2.217930913 |  |  |  |  |  |  |
|  |  |  |  | SPON1 | 2.770688931 |  |  |  |  |  |  |
|  |  |  |  | THBS1 | 1.032398423 |  |  |  |  |  |  |
|  |  |  |  | THBS3 | 1.500971066 |  |  |  |  |  |  |
|  |  |  |  | TMEFF2 | 3.061824421 |  |  |  |  |  |  |
|  |  |  |  | VWA1 | 1.601328164 |  |  |  |  |  |  |
