# Supplementary material for: Glis1 and oxaloacetate in nucleus pulposus stromal cell somatic reprogramming and survival
Source: Front Mol Biosci. 2022 Nov 3;9:1009402. doi: 10.3389/fmolb.2022.1009402 (PMC9671658; doi:10.3389/fmolb.2022.1009402)
Supplement: Supplementary file 3 [file Table2.DOCX]

**Supplementary Table 2:** Differentially expressed genes between annulus fibrosus (AF) and nucleus pulposus (NP) cells of the IVD and adipose (FAT) stromal cells of the same donor associated with the functional enrichment term DNA binding transcription factor activity (GO:0003700) displayed by the log2 fold changes (FC[log2]).

| DNA binding transcription factor activity (GO:0003700) | | | | | | | | | | | |
| --- | --- | --- | --- | --- | --- | --- | --- | --- | --- | --- | --- |
| NP-AF | | | | NP-FAT | | | | AF-FAT | | | |
| high in NP | FC[log2] | low in NP | FC[log2] | high in NP | FC[log2] | low in NP | FC[log2] | high in FAT | FC[log2] | Low in FAT | FC[log2] |
| ATF7 | 1.0424869 | ALX3 | 1.09070352 | ASCL4 | 1.445649773 | CEBPA | 1.443298203 | CEBPA | 1.212284138 | BARX1 | 2.595430233 |
| CSRNP1 | 1.0805314 | ARNT2 | 1.61145684 | ATF7 | 1.168813249 | DLX1 | 8.245695917 | DLX1 | 2.312382816 | E2F7 | 1.161973782 |
| DMRTA2 | 5.1724984 | ATF5 | 1.34563271 | BARX1 | 3.200854551 | DLX2 | 6.454510786 | DLX2 | 1.612746696 | ERG | 4.432813908 |
| EGR1 | 3.3891999 | DLX1 | 5.93022713 | BHLHE41 | 1.934892824 | DLX3 | 3.476133994 | DLX4 | 1.381705326 | FOXC2 | 4.941075041 |
| EGR2 | 4.0139337 | DLX2 | 4.85935511 | CEBPD | 2.277536935 | DLX4 | 3.129086177 | DMRTA2 | 4.335927655 | FOXD2 | 1.202910216 |
| EGR3 | 3.60517 | DLX3 | 3.09065861 | CIC | 1.153411832 | EPAS1 | 1.611321976 | EBF2 | 5.576790642 | FOXF1 | 3.993903669 |
| ERG | 1.46119 | DLX4 | 1.7430362 | CREB5 | 1.489443712 | GSC2 | 1.850344514 | FOXA3 | 1.2072111 | FOXF2 | 3.188034628 |
| FOS | 2.211755 | DMRT1 | 3.26888739 | DBP | 1.73567672 | HES1 | 2.247394882 | FOXE1 | 8.146587528 | FOXL1 | 8.797541634 |
| FOSB | 2.441437 | DMRT2 | 5.37844413 | E2F2 | 1.769573587 | HEYL | 3.325482016 | FOXG1 | 3.001532309 | FOXN1 | 10.40113186 |
| FOXA1 | 2.6017173 | ETV1 | 1.22417384 | E2F7 | 1.70635675 | HIC1 | 3.190734202 | HEYL | 3.233638325 | FOXP3 | 1.059486903 |
| FOXA3 | 2.2159301 | FOXN1 | 6.33125492 | E2F8 | 1.461988264 | HIVEP3 | 3.314843332 | HIVEP3 | 3.371054524 | GLI1 | 1.475881721 |
| FOXB1 | 7.6641189 | GATA2 | 4.78352402 | ELF3 | 1.874868069 | HOXA13 | 2.057720121 | HOXA13 | 1.148601764 | GLIS1 | 9.413087041 |
| FOXF2 | 1.120343 | GSC | 2.84052578 | ELK3 | 1.23686788 | HOXB4 | 1.355791083 | HOXB4 | 1.094326433 | HOXA1 | 2.12625647 |
| GLI2 | 6.562368 | GSC2 | 1.05461372 | ERG | 5.893225418 | HOXB9 | 1.68549092 | HOXB9 | 2.511441541 | HOXA2 | 5.688961669 |
| GLIS1 | 2.7883663 | HAND2 | 1.45360931 | ETS2 | 1.974929341 | HOXC5 | 1.281281532 | HOXC5 | 2.134859715 | HOXA3 | 2.402069116 |
| GLIS3 | 1.6030843 | HES1 | 1.61418158 | FLI1 | 1.09295996 | HOXC6 | 1.513359524 | HOXC6 | 1.459508558 | HOXA4 | 1.451374772 |
| GRHL1 | 1.8553544 | HES2 | 1.49579775 | FOXA1 | 2.865222796 | HOXC8 | 1.762937549 | HOXC8 | 2.377651909 | HOXA5 | 2.558906125 |
| HIF1A | 1.0412122 | HOXC11 | 1.17866558 | FOXA3 | 1.010159151 | IRX1 | 7.052418258 | IRX1 | 5.612317357 | HOXA6 | 2.159343058 |
| IKZF2 | 5.7923025 | HOXC12 | 1.39800273 | FOXB1 | 6.092273491 | ISL1 | 4.105375303 | ISL1 | 6.703371321 | HOXA7 | 1.128571209 |
| IRX2 | 6.7208848 | HOXC13 | 1.44704522 | FOXC2 | 5.234477941 | LHX3 | 1.346582692 | KLF7 | 1.666028451 | HOXB6 | 1.004673169 |
| ISL2 | 1.6870218 | KLF15 | 1.3254028 | FOXF1 | 4.189701335 | MEF2C | 1.245664744 | LHX3 | 1.689669446 | HOXC11 | 1.124849402 |
| JUN | 1.8090852 | KLF5 | 1.02497051 | FOXF2 | 4.306590793 | NHLH1 | 2.209286642 | MEF2C | 1.437869012 | HOXC12 | 4.966532619 |
| LMX1B | 2.3896335 | MEOX1 | 2.4098075 | FOXL1 | 8.682444277 | MEOX1 | 2.327958675 | MEIS1 | 1.894890898 | HOXC13 | 1.18959872 |
| MAF | 1.318216 | MEOX2 | 2.78160475 | FOXM1 | 1.456876712 | MSX1 | 5.025198377 | NFAT5 | 1.025994568 | HOXD10 | 5.161925742 |
| MEIS1 | 1.4782115 | MLXIPL | 2.44395217 | FOXO4 | 1.130562836 | MSX2 | 3.193596851 | NFATC2 | 1.520840501 | HOXD11 | 6.430889306 |
| NACC2 | 1.4866569 | MSC | 1.32033825 | FOXP2 | 1.110171729 | NR2E1 | 2.091659201 | NKX2-2 | 1.889614958 | HOXD9 | 1.630767307 |
| NFATC2 | 2.0178963 | MSX1 | 4.97447146 | FOXP3 | 1.694936136 | ONECUT2 | 6.767632112 | ONECUT2 | 5.898303731 | KLF13 | 1.392444818 |
| NKX2-2 | 4.2339834 | MSX2 | 2.43170435 | GLI1 | 1.520274215 | PAX5 | 6.325241507 | PAX2 | 3.50728366 | KLF15 | 1.984965038 |
| NKX2-8 | 1.0000423 | MYBL2 | 1.08965733 | GLI2 | 1.897380315 | RUNX2 | 2.483412986 | PAX5 | 5.350419156 | KLF5 | 1.098245405 |
| NKX3-2 | 3.4010598 | MYRF | 1.99493416 | GLIS1 | 12.19981621 | SATB2 | 1.193649809 | PAX8 | 2.228684862 | KLF9 | 1.662089758 |
| NPAS3 | 2.6925628 | NKX6-1 | 4.03399804 | GLIS3 | 2.46064281 | SHOX2 | 3.754701789 | RUNX2 | 1.968561953 | LMX1B | 2.42565736 |
| NR2F1 | 2.3796439 | NR2F2 | 1.25643657 | GRHL1 | 2.041017891 | SMAD6 | 1.054480789 | SHOX2 | 3.005176448 | MECOM | 2.436056372 |
| NR4A1 | 1.0145383 | NRL | 1.60026528 | HIF1A | 1.109218078 | SMAD7 | 1.219155236 | SOX2 | 7.246233351 | MEOX2 | 2.937808993 |
| NR4A2 | 1.4477893 | PAX3 | 1.82785516 | HLF | 1.548840467 | SP100 | 5.437159233 | SP100 | 4.036000042 | MNT | 1.543030231 |
| NTN1 | 1.0639745 | PAX6 | 1.24222699 | HNF1A | 2.199433189 | TBX15 | 4.813886736 | SP7 | 7.077292561 | MYBL1 | 2.112903904 |
| PAX2 | 3.7015275 | PBK | 1.80642971 | HOXA1 | 2.847646422 | TBX18 | 1.245289198 | TBX15 | 2.540298468 | NFATC1 | 1.478619019 |
| PAX8 | 2.1929985 | PBX3 | 1.056946 | HOXA2 | 5.67956076 | TBX2 | 4.762126438 | TBX18 | 1.893023863 | NFIB | 1.459127175 |
| PBX1 | 1.6038837 | PITX1 | 1.29137657 | HOXA3 | 2.213316192 | TBX20 | 1.413720244 | TBX20 | 1.73197758 | NKX2-8 | 6.423773199 |
| PTX3 | 1.9526799 | PRDM16 | 1.90161012 | HOXA4 | 1.596142483 | TBX21 | 4.997959217 | TBX21 | 6.834309245 | NKX3-2 | 5.157640393 |
| SALL1 | 8.9418387 | SIM2 | 1.65494291 | HOXA5 | 2.395115557 | TBX3 | 2.957158264 | TBX3 | 3.839895514 | NKX6-1 | 3.369987287 |
| SALL3 | 7.0502907 | SMAD6 | 1.18737721 | HOXA6 | 2.50144572 | TCF7 | 2.016474061 | TCF7 | 2.033662008 | NR2F2 | 1.341550467 |
| SATB1 | 1.2036203 | SOX12 | 2.13299449 | HOXA7 | 1.050918154 | TEC | 1.942821868 | TEC | 1.407521634 | NTN1 | 3.078973096 |
| SATB2 | 2.1436288 | TBX15 | 2.26868039 | HOXB6 | 1.001341558 | VDR | 2.113896801 | THRB | 1.473014525 | PAX1 | 9.702466092 |
| SOX10 | 1.3793342 | TBX2 | 4.06032709 | HOXC12 | 3.565922681 | ZFP37 | 1.442722977 | VDR | 1.894600765 | PAX3 | 2.233338575 |
| SOX2 | 7.2816955 | TP73 | 1.60769146 | HOXC4 | 1.693248601 | ZNF114 | 1.886503566 | ZFP37 | 1.853677367 | PAX6 | 1.014024909 |
| SOX5 | 1.8744434 | TWIST2 | 2.53385767 | HOXD10 | 4.825261113 | ZNF536 | 7.728235416 | ZNF114 | 1.030014146 | PAX9 | 5.03813122 |
| SOX6 | 2.2852806 | ZBTB7C | 1.69150413 | HOXD11 | 5.870711234 | ZNF597 | 1.07414784 | ZNF597 | 1.227656628 | PBX3 | 1.196136743 |
| SOX9 | 1.1054023 | ZBTB8A | 1.02414445 | HOXD3 | 1.097797952 |  |  |  |  | RARG | 1.298542366 |
| SP8 | 8.7868616 | ZIC1 | 1.32754598 | HOXD9 | 1.576681082 |  |  |  |  | SLC2A4RG | 1.607674404 |
| TBX1 | 3.3646741 | ZIC4 | 2.06726945 | IKZF2 | 1.307165367 |  |  |  |  | SOX12 | 3.331775236 |
| TBX10 | 2.3273732 | ZNF423 | 1.46334308 | IRF2 | 1.200527954 |  |  |  |  | SOX5 | 3.841415073 |
| THRB | 1.5339402 |  |  | IRF7 | 1.235823917 |  |  |  |  | SOX6 | 2.798402183 |
| TSHZ2 | 2.9665095 |  |  | IRF8 | 2.809348309 |  |  |  |  | SOX9 | 1.765189932 |
| ZFHX2 | 1.171653 |  |  | IRX2 | 6.582127341 |  |  |  |  | TCF4 | 1.235441801 |
| ZNF169 | 1.1427158 |  |  | IRX3 | 1.114563982 |  |  |  |  | ZNF362 | 1.232740068 |
| ZNF391 | 1.879003 |  |  | JUND | 1.15239048 |  |  |  |  |  |  |
| ZNF395 | 1.1367083 |  |  | KLF4 | 1.155624541 |  |  |  |  |  |  |
|  |  |  |  | KLF9 | 1.921114276 |  |  |  |  |  |  |
|  |  |  |  | LMX1B | 4.822324642 |  |  |  |  |  |  |
|  |  |  |  | MAFF | 2.384601353 |  |  |  |  |  |  |
|  |  |  |  | MECOM | 1.664806567 |  |  |  |  |  |  |
|  |  |  |  | MEIS3 | 1.209330992 |  |  |  |  |  |  |
|  |  |  |  | MXD3 | 1.425657374 |  |  |  |  |  |  |
|  |  |  |  | MXD4 | 1.187146425 |  |  |  |  |  |  |
|  |  |  |  | MXI1 | 1.360785204 |  |  |  |  |  |  |
|  |  |  |  | MYBL1 | 2.431164632 |  |  |  |  |  |  |
|  |  |  |  | MZF1 | 1.05668451 |  |  |  |  |  |  |
|  |  |  |  | NFATC1 | 1.711871766 |  |  |  |  |  |  |
|  |  |  |  | NFIA | 2.318726603 |  |  |  |  |  |  |
|  |  |  |  | NFIB | 2.437409384 |  |  |  |  |  |  |
|  |  |  |  | NFIX | 1.229675156 |  |  |  |  |  |  |
|  |  |  |  | NKX2-2 | 2.337089028 |  |  |  |  |  |  |
|  |  |  |  | NKX2-8 | 7.422555892 |  |  |  |  |  |  |
|  |  |  |  | NKX3-2 | 8.590355144 |  |  |  |  |  |  |
|  |  |  |  | NPAS3 | 7.589628601 |  |  |  |  |  |  |
|  |  |  |  | NR2C2 | 1.072897181 |  |  |  |  |  |  |
|  |  |  |  | NR2F6 | 1.182082049 |  |  |  |  |  |  |
|  |  |  |  | NR4A2 | 2.995712398 |  |  |  |  |  |  |
|  |  |  |  | NTN1 | 4.136505763 |  |  |  |  |  |  |
|  |  |  |  | OSR1 | 2.888310406 |  |  |  |  |  |  |
|  |  |  |  | PAX1 | 10.40422927 |  |  |  |  |  |  |
|  |  |  |  | PAX9 | 5.83210497 |  |  |  |  |  |  |
|  |  |  |  | PBX1 | 1.325123869 |  |  |  |  |  |  |
|  |  |  |  | PITX1 | 1.579969441 |  |  |  |  |  |  |
|  |  |  |  | PKNOX2 | 1.867160481 |  |  |  |  |  |  |
|  |  |  |  | POU2F1 | 1.067646459 |  |  |  |  |  |  |
|  |  |  |  | POU2F2 | 5.775806916 |  |  |  |  |  |  |
|  |  |  |  | PTX3 | 1.420648149 |  |  |  |  |  |  |
|  |  |  |  | RARG | 1.517920367 |  |  |  |  |  |  |
|  |  |  |  | RBPJL | 1.55248447 |  |  |  |  |  |  |
|  |  |  |  | REL | 4.071075049 |  |  |  |  |  |  |
|  |  |  |  | RELB | 1.28450249 |  |  |  |  |  |  |
|  |  |  |  | RFX3 | 2.237554162 |  |  |  |  |  |  |
|  |  |  |  | SALL1 | 6.377407487 |  |  |  |  |  |  |
|  |  |  |  | SALL3 | 6.911635419 |  |  |  |  |  |  |
|  |  |  |  | SCX | 2.514585418 |  |  |  |  |  |  |
|  |  |  |  | SIM2 | 7.528205041 |  |  |  |  |  |  |
|  |  |  |  | SIX1 | 2.336080099 |  |  |  |  |  |  |
|  |  |  |  | SIX4 | 2.264533402 |  |  |  |  |  |  |
|  |  |  |  | SIX5 | 1.288871733 |  |  |  |  |  |  |
|  |  |  |  | SLC2A4RG | 1.439742851 |  |  |  |  |  |  |
|  |  |  |  | SOX10 | 1.528827825 |  |  |  |  |  |  |
|  |  |  |  | SOX12 | 1.196096484 |  |  |  |  |  |  |
|  |  |  |  | SOX4 | 1.143367826 |  |  |  |  |  |  |
|  |  |  |  | SOX5 | 5.704809891 |  |  |  |  |  |  |
|  |  |  |  | SOX6 | 5.081841515 |  |  |  |  |  |  |
|  |  |  |  | SOX9 | 2.872875366 |  |  |  |  |  |  |
|  |  |  |  | SP8 | 8.648293371 |  |  |  |  |  |  |
|  |  |  |  | SREBF2 | 1.193991824 |  |  |  |  |  |  |
|  |  |  |  | STAT4 | 1.104101411 |  |  |  |  |  |  |
|  |  |  |  | STC2 | 1.28984897 |  |  |  |  |  |  |
|  |  |  |  | STK32B | 7.526871712 |  |  |  |  |  |  |
|  |  |  |  | STK33 | 2.566697226 |  |  |  |  |  |  |
|  |  |  |  | STK38L | 1.381481067 |  |  |  |  |  |  |
|  |  |  |  | STMN1 | 1.268171631 |  |  |  |  |  |  |
|  |  |  |  | STX1B | 2.125493265 |  |  |  |  |  |  |
|  |  |  |  | STXBP5 | 1.149024752 |  |  |  |  |  |  |
|  |  |  |  | STXBP6 | 2.665622985 |  |  |  |  |  |  |
|  |  |  |  | SULF1 | 1.363644707 |  |  |  |  |  |  |
|  |  |  |  | SULF2 | 2.255769564 |  |  |  |  |  |  |
|  |  |  |  | TBX1 | 7.886796397 |  |  |  |  |  |  |
|  |  |  |  | TCF4 | 1.999959697 |  |  |  |  |  |  |
|  |  |  |  | TFAP4 | 1.856726081 |  |  |  |  |  |  |
|  |  |  |  | TGIF1 | 1.577091332 |  |  |  |  |  |  |
|  |  |  |  | TP53 | 1.203763897 |  |  |  |  |  |  |
|  |  |  |  | TP73 | 1.889256453 |  |  |  |  |  |  |
|  |  |  |  | TSHZ3 | 1.032540229 |  |  |  |  |  |  |
|  |  |  |  | VWA1 | 1.601328164 |  |  |  |  |  |  |
|  |  |  |  | ZBTB7C | 6.827514424 |  |  |  |  |  |  |
|  |  |  |  | ZCWPW1 | 1.455556853 |  |  |  |  |  |  |
|  |  |  |  | ZFHX3 | 1.337564336 |  |  |  |  |  |  |
|  |  |  |  | ZFP3 | 1.011696009 |  |  |  |  |  |  |
|  |  |  |  | ZIC1 | 2.583111176 |  |  |  |  |  |  |
|  |  |  |  | ZIC4 | 2.169866369 |  |  |  |  |  |  |
|  |  |  |  | ZNF331 | 1.306601777 |  |  |  |  |  |  |
|  |  |  |  | ZNF362 | 1.353094732 |  |  |  |  |  |  |
|  |  |  |  | ZNF367 | 1.724633216 |  |  |  |  |  |  |
|  |  |  |  | ZNF385B | 6.426638985 |  |  |  |  |  |  |
|  |  |  |  | ZNF395 | 3.514727537 |  |  |  |  |  |  |
|  |  |  |  | ZNF580 | 1.009438538 |  |  |  |  |  |  |
|  |  |  |  | ZNF581 | 1.274626732 |  |  |  |  |  |  |
|  |  |  |  | ZNF584 | 1.805399441 |  |  |  |  |  |  |
|  |  |  |  | ZNF608 | 8.408820831 |  |  |  |  |  |  |
|  |  |  |  | ZNF619 | 1.275901658 |  |  |  |  |  |  |
|  |  |  |  | ZNF652 | 1.236698618 |  |  |  |  |  |  |
|  |  |  |  | ZNF692 | 1.071958597 |  |  |  |  |  |  |
|  |  |  |  | ZNF853 | 3.452568063 |  |  |  |  |  |  |
|  |  |  |  | ZSCAN21 | 1.025956193 |  |  |  |  |  |  |
