# Supplementary material for: Glis1 and oxaloacetate in nucleus pulposus stromal cell somatic reprogramming and survival
Source: Front Mol Biosci. 2022 Nov 3;9:1009402. doi: 10.3389/fmolb.2022.1009402 (PMC9671658; doi:10.3389/fmolb.2022.1009402)
Supplement: Supplementary file 4 [file Table5.DOCX]

**Supplementary Table 5:** Differentially expressed genes between annulus fibrosus (AF) and nucleus pulposus (NP) cells of the IVD and adipose (FAT) stromal cells of the same donor associated with metalloproteinases displayed by the log2 fold changes (FC[log2]).

| Metalloproteinase associated | | | | | | | | | | | |
| --- | --- | --- | --- | --- | --- | --- | --- | --- | --- | --- | --- |
| AF-NP | | | | FAT-NP | | | | FAT-AF | | | |
| high in NP | FC[log2] | low in NP | FC[log2] | high in NP | FC[log2] | low in NP | FC[log2] | high in FAT | FC[log2] | low in FAT | FC[log2] |
| ADAM23 | 6.469194166 | ADAMTS20 | 2.292300363 | ADAM23 | 4.8348082 | ADAM19 | 2.983457635 | ADAM10 | 1.100663944 | ADAM8 | 1.090572461 |
| ADAMDEC1 | 6.932213493 | ADAMTSL1 | 4.284282674 | ADAMDEC1 | 6.793816217 | ADAMTS1 | 1.844992258 | ADAM19 | 2.362796216 | ADAMTS16 | 3.450056879 |
| ADAMTS1 | 2.161438844 | MMP1 | 2.052753826 | ADAMTS16 | 4.46062112 | ADAMTS14 | 5.843568247 | ADAMTS1 | 4.004657198 | ADAMTS17 | 1.149450206 |
| ADAMTS12 | 1.047346433 | MMP13 | 1.438775369 | ADAMTS17 | 2.91531699 | ADAMTS20 | 2.712689954 | ADAMTS14 | 5.449528951 | ADAMTS3 | 1.34891703 |
| ADAMTS16 | 1.01148384 | MMP25 | 3.936001925 | ADAMTS3 | 1.083362449 | ADAMTS5 | 2.520766473 | ADAMTS5 | 2.497622213 | MMP13 | 4.98308193 |
| ADAMTS17 | 1.769586266 |  |  | ADAMTSL2 | 2.525068336 | ADAMTS7 | 1.655864948 | ADAMTS6 | 1.573750149 | MMP3 | 6.264397247 |
| ADAMTSL2 | 2.301526963 |  |  | ADAMTSL4 | 1.780763126 | ADAMTS9 | 1.403897161 | ADAMTS7 | 2.477784209 |  |  |
| ADAMTSL4 | 1.768060367 |  |  | MMP13 | 3.525052616 | ADAMTSL1 | 6.018575288 | ADAMTS9 | 1.460041218 |  |  |
| MMP16 | 1.41490635 |  |  | MMP16 | 2.15906534 | ADAMTSL3 | 1.152282359 | ADAMTSL1 | 1.732762351 |  |  |
| MMP23B | 1.550601039 |  |  | MMP3 | 6.32003763 | MMP1 | 2.116471467 | MMP11 | 1.546791988 |  |  |
|  |  |  |  |  |  | MMP12 | 8.205898976 | MMP12 | 5.281493467 |  |  |
|  |  |  |  |  |  | MMP15 | 1.081448335 | MMP17 | 3.034137168 |  |  |
|  |  |  |  |  |  | MMP17 | 3.145713902 | MMP19 | 4.060851203 |  |  |
|  |  |  |  |  |  | MMP19 | 3.957872574 | MMP23B | 5.711153544 |  |  |
|  |  |  |  |  |  | MMP2 | 1.394548989 | MMP9 | 2.284880502 |  |  |
|  |  |  |  |  |  | MMP23B | 4.166329493 |  |  |  |  |
|  |  |  |  |  |  | MMP25 | 4.591285698 |  |  |  |  |
|  |  |  |  |  |  | MMP9 | 6.31687025 |  |  |  |  |
