# Supplementary material for: Glis1 and oxaloacetate in nucleus pulposus stromal cell somatic reprogramming and survival
Source: Front Mol Biosci. 2022 Nov 3;9:1009402. doi: 10.3389/fmolb.2022.1009402 (PMC9671658; doi:10.3389/fmolb.2022.1009402)
Supplement: Supplementary file 5 [file Table3.DOCX]

**Supplementary Table 3:** Differentially expressed genes between annulus fibrosus (AF) and nucleus pulposus (NP) cells of the IVD and adipose (FAT) stromal cells of the same donor associated with the functional enrichment term signaling receptor regulator activity (GO:0030545) displayed by the log2 fold changes (FC[log2]).

| Signaling receptor regulator activity (GO:0030545) | | | | | | | | | | | |
| --- | --- | --- | --- | --- | --- | --- | --- | --- | --- | --- | --- |
| AF-NP | | | | FAT-NP | | | | FAT-AF | | | |
| high in NP | FC[log2] | low in NP | FC[log2] | high in NP | FC[log2] | low in NP | FC[log2] | high in FAT | FC[log2] | low in FAT | FC[log2] |
| ANGPTL8 | 1.7589702 | ADIPOQ | 1.91455067 | ADIPOQ | 1.261953294 | ADCYAP1 | 10.0862312 | ADCYAP1 | 10.0171632 | ADIPOQ | 3.184899876 |
| APLN | 2.0650141 | APOA1 | 1.1714696 | ADM | 3.692179544 | AGRN | 1.29764905 | ANGPT4 | 1.95083457 | ADM | 2.723005143 |
| BMP2 | 1.1960546 | BDNF | 3.03716393 | ANGPTL8 | 8.274945951 | ANGPT4 | 2.01601548 | ARTN | 6.1181968 | ANGPTL8 | 6.52377959 |
| BTC | 4.2636113 | C5 | 2.70859276 | APLN | 2.387647911 | ARTN | 6.29795541 | BDNF | 1.82651044 | APOA1 | 3.968767494 |
| CCL2 | 4.851765 | CDC42EP2 | 1.57472583 | APOA1 | 2.795908091 | BDNF | 4.86610479 | BMP1 | 1.21792348 | APP | 1.112509314 |
| DIO2 | 4.4698929 | CNTF | 1.15687035 | APP | 1.46598742 | CCL17 | 4.54682106 | CCL17 | 5.1986663 | BMP4 | 1.427386126 |
| DKK1 | 3.4650775 | CSF1 | 1.10033473 | BMP2 | 1.072645468 | CCL2 | 4.14931696 | CCL2 | 8.99887549 | C5 | 6.692631486 |
| DKK2 | 2.5133561 | CXCL12 | 2.62791378 | BMP4 | 1.999086069 | CCL26 | 8.34642742 | CCL26 | 8.27468688 | CCL20 | 6.467485799 |
| DPP4 | 1.0447635 | CXCL5 | 2.69972955 | BTC | 8.563290818 | CDC42EP2 | 3.0758046 | CDC42EP2 | 1.49822157 | CCL28 | 1.813930137 |
| EFEMP1 | 3.3330155 | EPHA7 | 2.16619633 | CCL20 | 6.866785108 | CRLF1 | 7.66898253 | CRLF1 | 6.72868928 | CLCF1 | 1.641252499 |
| FGF13 | 5.3981789 | FGF16 | 5.41701061 | CCL28 | 2.599854045 | CSF1 | 3.15232376 | CSF1 | 2.0497003 | CLEC11A | 1.115971234 |
| FGF14 | 1.8670753 | FGF18 | 1.83967601 | CSPG5 | 1.115234532 | CXCL12 | 4.18805644 | CXCL12 | 1.56307773 | CXCL14 | 7.095741864 |
| FGF22 | 1.1540371 | FGF7 | 1.01945241 | CXCL14 | 7.574946414 | CXCL5 | 4.47084862 | CXCL5 | 1.76820194 | CXCL3 | 1.681269191 |
| FGFR2 | 1.9300619 | FGF9 | 4.82192848 | CXCL3 | 1.117028614 | CXCL8 | 4.58873836 | CXCL8 | 4.32631547 | DPP4 | 2.524933364 |
| FGFR3 | 1.8394521 | IL1RN | 3.20967545 | DIO2 | 6.074491946 | FGF1 | 3.87109684 | FBN1 | 1.57566991 | EFNA5 | 3.805798537 |
| GAL | 6.5093167 | IL21R | 4.21901844 | DKK1 | 8.085241157 | FGF18 | 6.05295776 | FGF1 | 3.57119756 | EPHA7 | 5.672020167 |
| GAS6 | 1.9304839 | IL34 | 1.3840267 | DPP4 | 3.568327036 | FGF19 | 8.0873847 | FGF18 | 4.22967751 | FGF10 | 1.687338555 |
| GDF5 | 8.1187097 | IL36A | 1.32543718 | EFEMP1 | 3.998377154 | FGF2 | 1.38537418 | FGF19 | 6.16711585 | FGF14 | 2.233510604 |
| GDF6 | 5.9968385 | IL36RN | 1.00818805 | EFNA5 | 3.263580341 | FGF7 | 1.07323895 | FGF2 | 1.09799701 | FGF16 | 4.041897926 |
| HBEGF | 1.2038747 | MSTN | 1.5772076 | FGF13 | 6.261698437 | FST | 1.5549638 | GAL | 5.51666924 | FLRT2 | 1.025534257 |
| IGF2 | 8.7588003 | OSTN | 5.33340025 | FGF14 | 4.082884718 | GDF15 | 1.89493201 | GDF11 | 1.2561435 | FLT3LG | 2.409895324 |
| IL11 | 1.0486528 | PMCH | 1.07433413 | FGF22 | 1.320551769 | GRP | 8.73654922 | GDF15 | 2.05701632 | GAS6 | 4.045116248 |
| IL6 | 2.7092994 | PTN | 2.03482614 | FGFR2 | 1.195492797 | HBEGF | 1.78653429 | GDNF | 1.2752187 | HDGF | 1.156782887 |
| INHBE | 1.8165442 | SEMA4A | 1.66202911 | FGFR3 | 2.101833959 | IL12A | 5.26109532 | GRP | 7.08126358 | HGF | 1.396265953 |
| JAG1 | 1.4234694 | SEMA5B | 1.51457278 | GAS6 | 5.975430089 | IL18 | 3.17733127 | HBEGF | 2.99028902 | HMGB1 | 1.746023274 |
| JAG2 | 2.2898959 | SEMA6A | 1.3515037 | GDF5 | 8.942110948 | IL1RN | 2.45443126 | IL12A | 3.08243631 | HMGB2 | 2.288635226 |
| LIF | 3.6679379 | SFRP2 | 2.16698621 | GDF6 | 6.276863152 | IL36A | 2.45919032 | IL18 | 2.37475463 | IL34 | 1.417536367 |
| LYVE1 | 3.4281044 | SST | 5.21324076 | GREM1 | 1.319476296 | IL36B | 1.41712305 | IL36A | 1.13150518 | IL6 | 3.530604459 |
| MIA | 3.8638409 | TNFSF15 | 2.09948137 | HGF | 2.256026125 | IL36RN | 1.3236151 | INHA | 1.97710187 | INHBE | 1.642486645 |
| NDP | 1.4229217 | TTR | 1.41625938 | HMGB1 | 1.255887032 | INHA | 1.44831966 | JAG1 | 3.04381196 | MIA | 5.48431378 |
| NRG2 | 1.700834 | VGF | 1.28337427 | HMGB2 | 1.908376801 | JAG1 | 1.62196185 | KITLG | 2.52743725 | MIF | 1.29464957 |
| NTN1 | 1.0639745 |  |  | IGF2 | 5.452402705 | KITLG | 3.00626653 | LIF | 3.44013072 | NDP | 1.216852292 |
| OGN | 3.6067065 |  |  | IL17D | 1.444516229 | LY6G6E | 1.7169571 | LY6G6E | 2.0409531 | NTN1 | 3.078973096 |
| PDGFC | 1.3935619 |  |  | IL6 | 6.232518233 | MARK1 | 1.9182403 | MARK1 | 1.37139685 | OGN | 2.083623786 |
| PGLYRP1 | 1.9090925 |  |  | INHBE | 3.451113552 | MDK | 1.56639548 | PDYN | 2.31494198 | OSTN | 3.824125272 |
| SEMA3A | 2.5344467 |  |  | JAG2 | 2.039155936 | MRAP2 | 2.82458286 | PGLYRP1 | 2.58776937 | PMCH | 1.026429709 |
| SEMA3B | 1.3012662 |  |  | MIA | 9.356514062 | PDYN | 3.16448445 | SEMA3E | 1.16869907 | SCG2 | 2.54460772 |
| SEMA3E | 2.2645438 |  |  | MIF | 1.720942657 | PTHLH | 1.11537355 | SEMA3F | 1.69248432 | SEMA3B | 2.708045692 |
| SEMA4B | 2.0885891 |  |  | NDP | 2.639576513 | PTN | 1.27871 | SEMA4B | 1.71557246 | SEMA3C | 1.377239734 |
| SEMA4D | 2.0565692 |  |  | NRG2 | 5.163932183 | SEMA3F | 1.14150808 | SEMA5A | 8.0963842 | SEMA4A | 1.416297744 |
| SEMA5A | 5.5331202 |  |  | NRTN | 2.421369744 | SEMA5A | 2.56569109 | SEMA6B | 3.50263549 | SEMA4D | 2.556237207 |
| STC2 | 1.6854015 |  |  | NTN1 | 4.136505763 | SEMA6B | 3.74110036 | SEMA6D | 1.86006049 | SEMA6A | 3.647452917 |
| TAC4 | 1.3916056 |  |  | OGN | 5.689254872 | SEMA6D | 1.10437547 | SEMA7A | 4.63489433 | SPP1 | 1.178517311 |
| TG | 2.9579002 |  |  | PROM2 | 1.705096573 | SEMA7A | 4.32957407 | SFRP2 | 5.72062064 | SST | 1.28955409 |
| TGFA | 4.486613 |  |  | SEMA3A | 1.53924625 | SFRP2 | 7.90880979 | SLIT2 | 7.39584719 | TG | 3.309288078 |
| TGFBR2 | 1.4245692 |  |  | SEMA3B | 4.007388784 | SLIT2 | 3.80713062 | TGFA | 2.29648404 | TNFRSF11B | 6.006632639 |
| TNFRSF11B | 2.8934419 |  |  | SEMA3C | 1.976112337 | SLIT3 | 5.4597982 | TSLP | 2.33531253 | TNFSF15 | 1.944228155 |
| WNT2B | 1.0391373 |  |  | SEMA3E | 1.096795962 | SPX | 1.77705808 | VEGFA | 1.59474782 | VEGFB | 1.333846243 |
| WNT5A | 3.0107206 |  |  | SEMA4D | 4.60190486 | SST | 3.92596382 | VEGFC | 2.00108717 |  |  |
|  |  |  |  | SEMA6A | 2.290147973 | TIMP1 | 1.29684133 | VGF | 4.48316683 |  |  |
|  |  |  |  | SPP1 | 1.683367832 | TSLP | 1.74205543 | WNT10B | 1.81450359 |  |  |
|  |  |  |  | STC2 | 1.28984897 | TTR | 1.16369661 | WNT11 | 9.62537457 |  |  |
|  |  |  |  | TAC4 | 1.453704679 | VEGFA | 1.00390655 | WNT2 | 5.10394603 |  |  |
|  |  |  |  | TG | 6.285366774 | VEGFC | 1.87001989 | WNT5A | 4.48173959 |  |  |
|  |  |  |  | TGFA | 2.187287877 | VGF | 5.76532073 | WNT5B | 1.32941737 |  |  |
|  |  |  |  | TNFRSF11B | 8.901680534 | WNT10B | 2.64617655 | ZNF106 | 1.2861103 |  |  |
|  |  |  |  | TNFSF13B | 4.77027075 | WNT11 | 8.95987265 |  |  |  |  |
|  |  |  |  | TNFSF18 | 1.249992759 | WNT2 | 4.16878082 |  |  |  |  |
|  |  |  |  |  |  | WNT5A | 1.47224968 |  |  |  |  |
|  |  |  |  |  |  | WNT5B | 1.84116816 |  |  |  |  |
|  |  |  |  |  |  | WNT9A | 1.33654418 |  |  |  |  |
|  |  |  |  |  |  | ZNF106 | 1.3910277 |  |  |  |  |
